# Supplementary material for: Inter-annual variation in seasonal dengue epidemics driven by multiple interacting factors in Guangzhou, China
Source: Nat Commun. 2019 Mar 8;10:1148. doi: 10.1038/s41467-019-09035-x (PMC6408462; doi:10.1038/s41467-019-09035-x)
Supplement: Supplementary file 1 — Supplementary Information [file 41467_2019_9035_MOESM1_ESM.pdf]

Supplementary Information for

Inter-annual variation in seasonal dengue epidemics driven by multiple interacting factors in Guangzhou, China

Rachel J. Oidtman[1], Shengjie Lai[2,3,4], Zhoujie Huang[2], Juan Yang[2], Amir S. Siraj[1], Robert C. Reiner[5], Andrew J. Tatem[3,4], T. Alex Perkins*[1], Hongjie Yu*[2]

[1] Department of Biological Sciences and Eck Institute for Global Health, University of Notre Dame, Notre Dame, IN, USA
[2] School of Public Health, Fudan University, Key Laboratory of Public Health Safety, Ministry of Education, Shanghai, China
[3] WorldPop, Department of Geography and Environment, University of Southampton, Southampton, UK
[4] Flowminder Foundation, Stockholm, Sweden
[5] Institute for Health and Metrics and Evaluation, University of Washington, Seattle, WA, USA

* Corresponding authors: yhj@fudan.edu.cn and taperkins@nd.edu

SUPPLEMENTARY TABLES

Paper	Location	Time step	Time frame	Type of model	Factors / covariates included	Main conclusion
1	Guangzhou	Monthly	2005-2015	Statistical	Avg. temperature, avg. min. temperature, avg. max. temperature, accumulated precipitation, number of days with rainfall, MOI, and BI	Climate conditions explain temporal dynamics of dengue incidence
2	Guangzhou	Monthly	2006-Sept. 2014	Statistical	Local min. temperature, accumulative precipitation	Minimum temperature in previous month and accumulative precipitation with 3-month lag can project dengue outbreaks of 2013 and 2014
3	Guangdong	Weekly	2014	Mechanistic	Weekly avg. temperature, and weekly precipitation	Delayed mosquito control, continuous importations from April-July, transmission of asymptomatic infections, and high precipitation from May-August are causal factors for unprecedented outbreak
4	Guangzhou	Daily	2013-2014	Mechanistic, deterministic	Temperature, rainfall, and evaporation	In 2013 and 2014, date of first imported case and unusually high precipitation, and in 2014 only, delayed interventions and vertical transmission, are factors responsible for patterns of moderate outbreak in 2013 and much larger outbreak in 2014
5	Guangzhou	Daily	2014	Spatial	Urbanization level, ratio of urban village, road density, population density, GDP, NDVI, temperature, and precipitation	Temperature, precipitation, road density, and water body area were the dominant factors affecting dengue virus transmission in the 2014 outbreak
6	Guangzhou	Daily	Sept. 2014-Nov. 2014	Mechanistic	Population density, human mobility (transportation), temperature, rainfall, humidity, MOI, and BI	Urbanization, vector activities, and human behavior played significant roles in shaping the 2014 dengue outbreak and the patterns of its spread
7	Guangzhou	Daily	2013-2014	Mechanistic, stochastic	Tourist exchange, temperature, precipitation, BI, and MOI	Higher number of imported cases in May and June were the most important determinants of dengue outbreaks

Supplementary Table 1. Model attributes, covariates included, and conclusions of papers that investigated dengue incidence in Guangzhou or Guangdong in 2014 and other years.

Parameter	Point estimate	Upper C.I.
$\theta_{s_{m_1}}$	1.03	1.08
$\theta_{s_{m_2}}$	1.03	1.09
$\theta_{s_{m_3}}$	1.03	1.10
$\theta_{s_{m_4}}$	1.01	1.04
$\theta_{s_{m_5}}$	1.03	1.11
$\theta_{s_{m_6}}$	1.03	1.08
$\theta_{s_{m_7}}$	1.00	1.01
$\theta_{s_{m_8}}$	1.00	1.00
$\theta_{s_{m_9}}$	1.00	1.00
$\theta_{s_{T_1}}$	1.04	1.10
$\theta_{s_{T_2}}$	1.03	1.09
$\theta_{s_{T_3}}$	1.03	1.09
$\theta_{s_{T_4}}$	1.04	1.12
$\theta_{s_{T_5}}$	1.02	1.07
$\theta_{s_{T_6}}$	1.02	1.07
$\theta_{s_{T_7}}$	1.02	1.06
$\theta_{s_{T_8}}$	1.00	1.01
$\theta_{s_{T_9}}$	1.00	1.01
θ_{0_1}	1.05	1.15
θ_{0_2}	1.01	1.01
θ_{0_3}	1.15	1.45
θ_{0_4}	1.03	1.07
θ_{0_5}	1.05	1.18
θ_{0_6}	1.06	1.19
θ_{0_7}	1.04	1.14
θ_{0_8}	1.04	1.12
θ_{0_9}	1.09	1.28
$\theta_{0_{10}}$	1.06	1.17
$\theta_{0_{11}}$	1.03	1.08
$\theta_{0_{12}}$	1.01	1.02
$\theta_{0_{13}}$	1.05	1.16
$\theta_{0_{14}}$	1.00	1.01
$\theta_{0_{15}}$	1.02	1.06
$\theta_{0_{16}}$	1.19	1.56
$\theta_{0_{17}}$	1.04	1.05
$\theta_{0_{18}}$	1.09	1.18
$\theta_{0_{19}}$	1.04	1.12
$\theta_{0_{20}}$	1.03	1.08
$\theta_{0_{21}}$	1.06	1.17
$\theta_{0_{22}}$	1.09	1.21

$\theta_{0_{23}}$	1.03	1.07
$\theta_{0_{24}}$	1.04	1.08
$\theta_{0_{25}}$	1.04	1.14
$\theta_{0_{26}}$	1.04	1.09
$\theta_{0_{27}}$	1.02	1.07
$\theta_{0_{28}}$	1.03	1.05
$\theta_{0_{29}}$	1.05	1.17
$\theta_{0_{30}}$	1.03	1.06
$\theta_{0_{31}}$	1.10	1.31
$\theta_{0_{32}}$	1.03	1.10
$\theta_{0_{33}}$	1.16	1.46

Supplementary Table 2. Gelman-Rubin convergence diagnostic based on three independent Sequential Monte Carlo (SMC) sampling routines. Convergence is diagnosed to have occurred when the upper confidence interval is close to 1. The multivariate potential scale reduction factor is 1.72.

SUPPLEMENTARY FIGURES

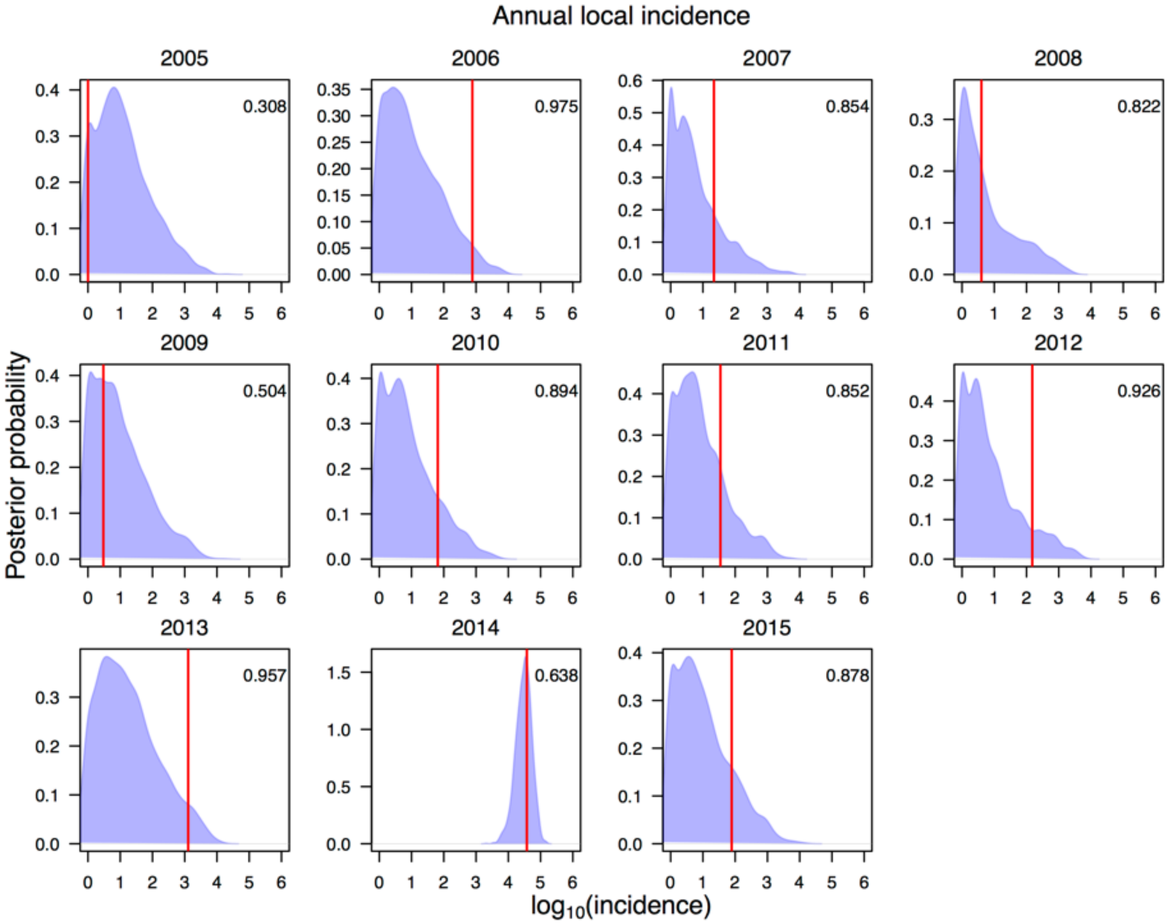

Supplementary Fig. 1. Posterior predictive distribution of total dengue incidence for each year based on 2,000 simulations from the fitted transmission model. The red line shows observed annual local incidence for each year. The number in each panel indicates the Bayesian p-value, with values between 0.025 and 0.975 indicating model consistency with the data.

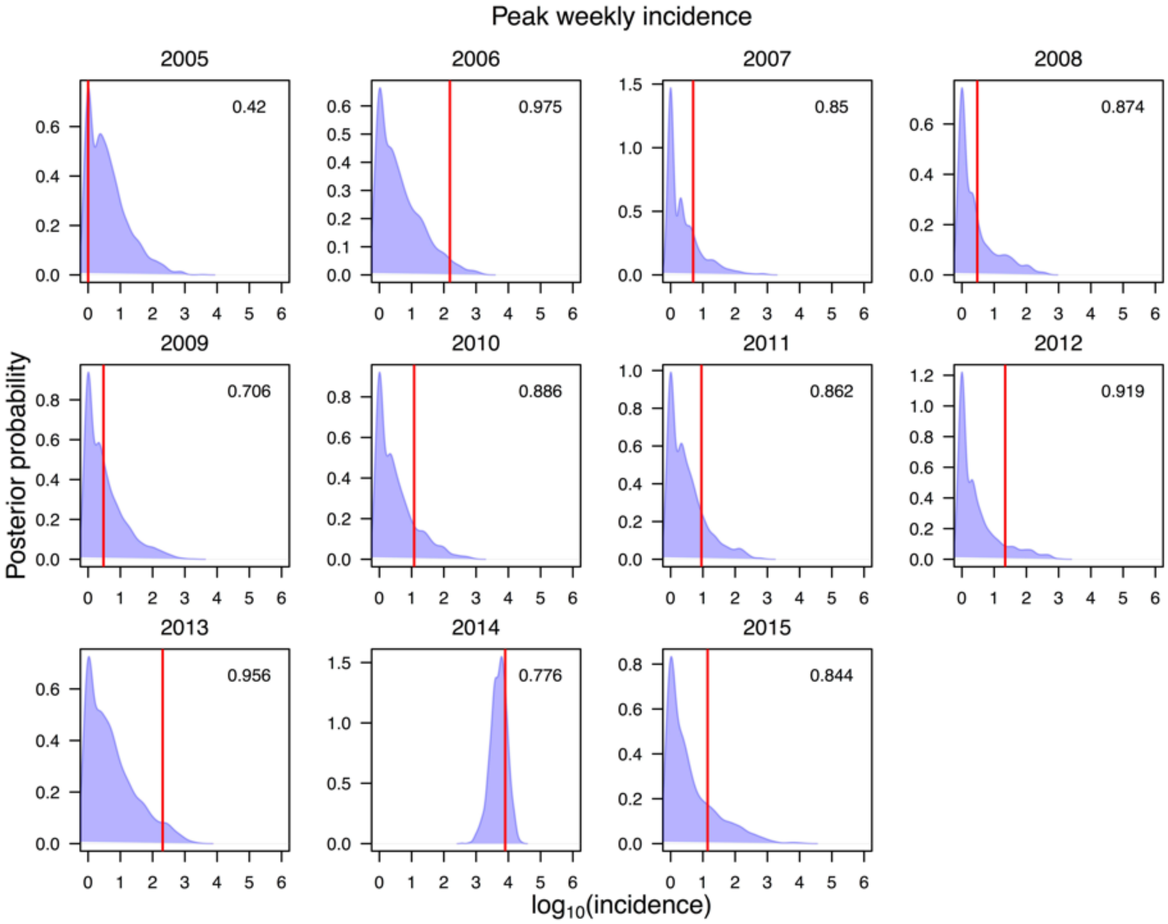

Supplementary Fig. 2. Posterior predictive distribution of peak weekly incidence for each year based on 2,000 simulations from the fitted transmission model. The red line shows observed peak weekly dengue incidence for each year. The number in each panel indicates the Bayesian p-value, with values between 0.025 and 0.975 indicating model consistency with the data.

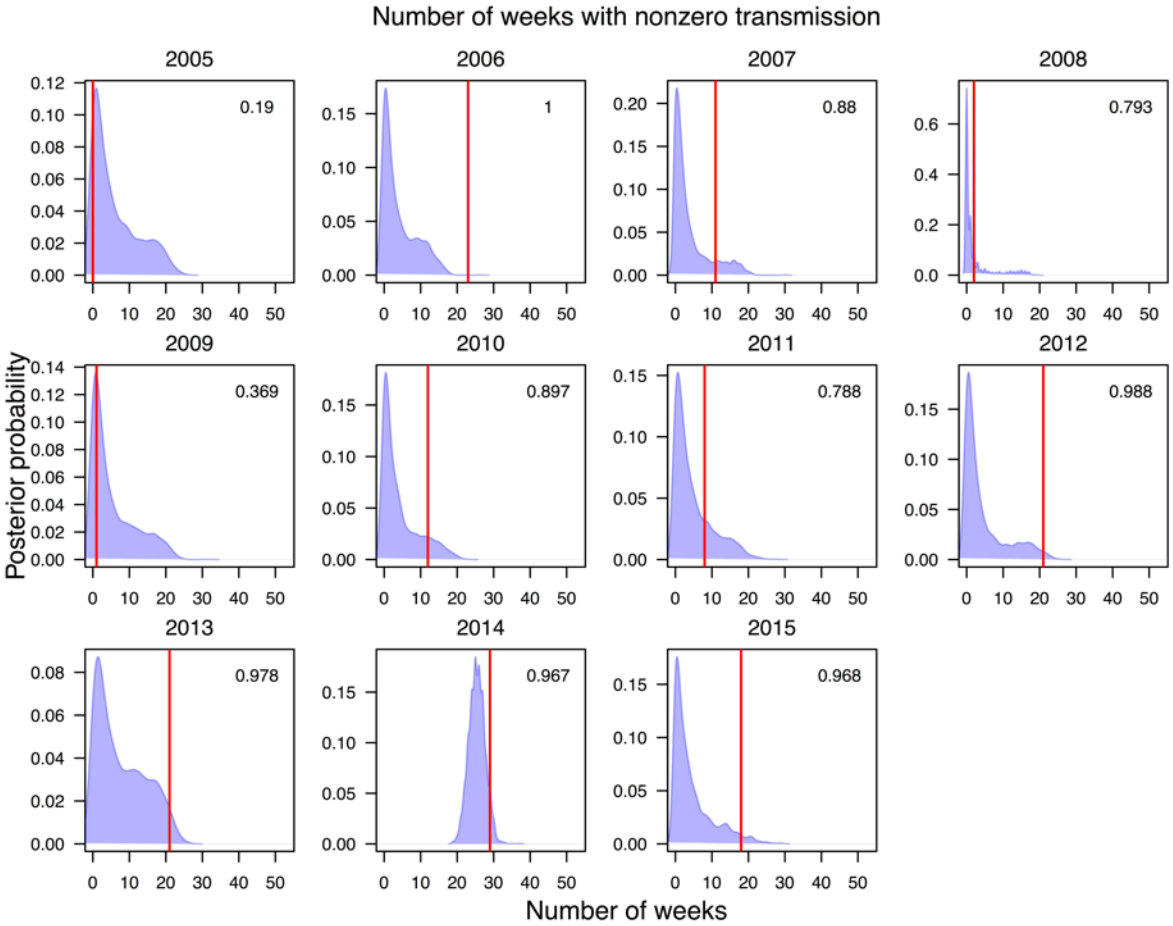

Supplementary Fig. 3. Posterior predictive distribution of the number of weeks with non-zero dengue incidence in each year based on 2,000 simulations from the fitted transmission model. The red line shows observed number of weeks with non-zero dengue incidence for each year. The number in each panel indicates the Bayesian p-value, with values between 0.025 and 0.975 indicating model consistency with the data.

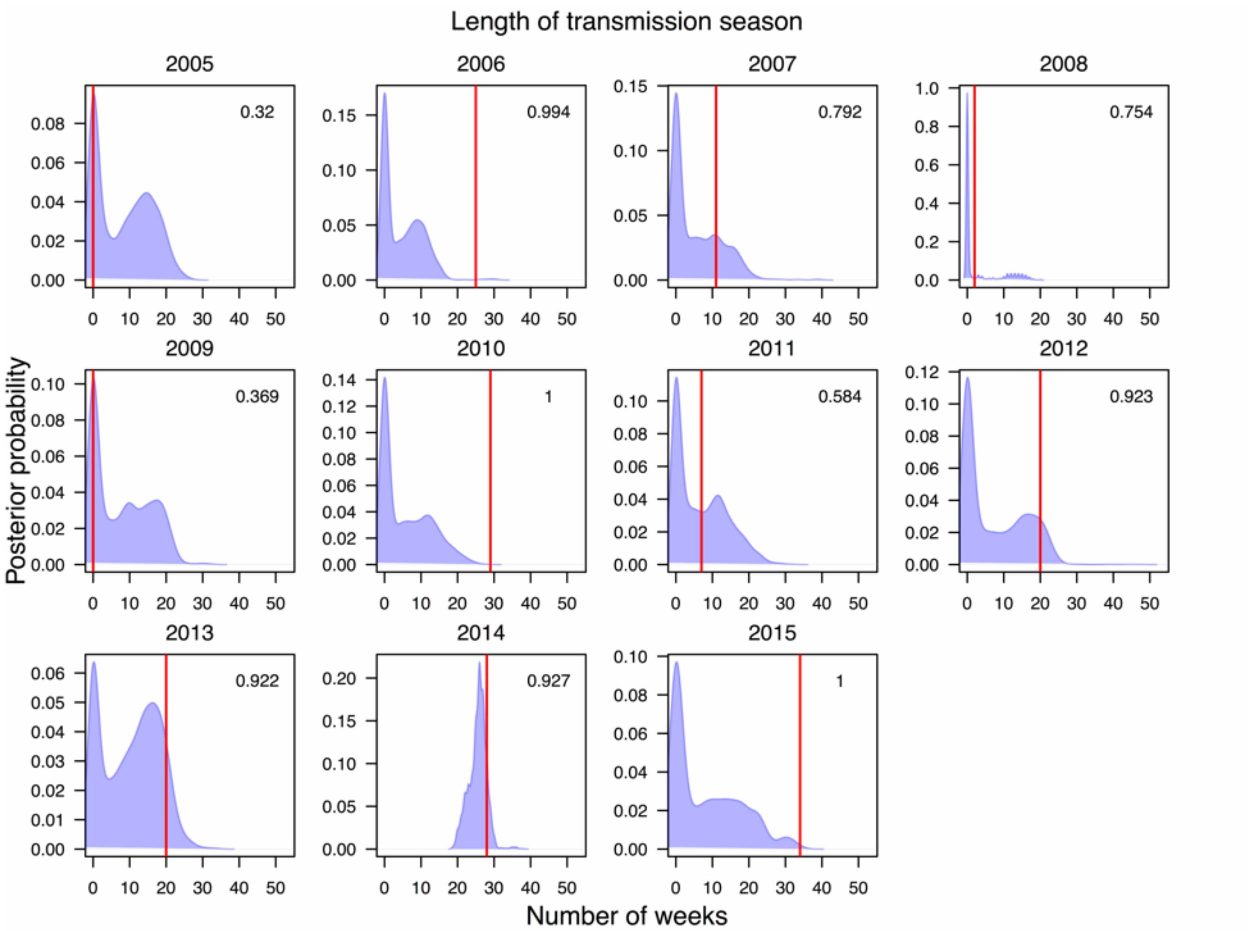

Supplementary Fig. 4. Posterior predictive distribution of the difference between the first non-zero dengue incidence week to the last non-zero dengue incidence week in each year based on 2,000 simulations from the fitted transmission model. The red line shows observed difference between the first non-zero dengue incidence week to the last non-zero dengue incidence day for each year. The number in each panel indicates the Bayesian p-value, with values between 0.025 and 0.975 indicating model consistency with the data.

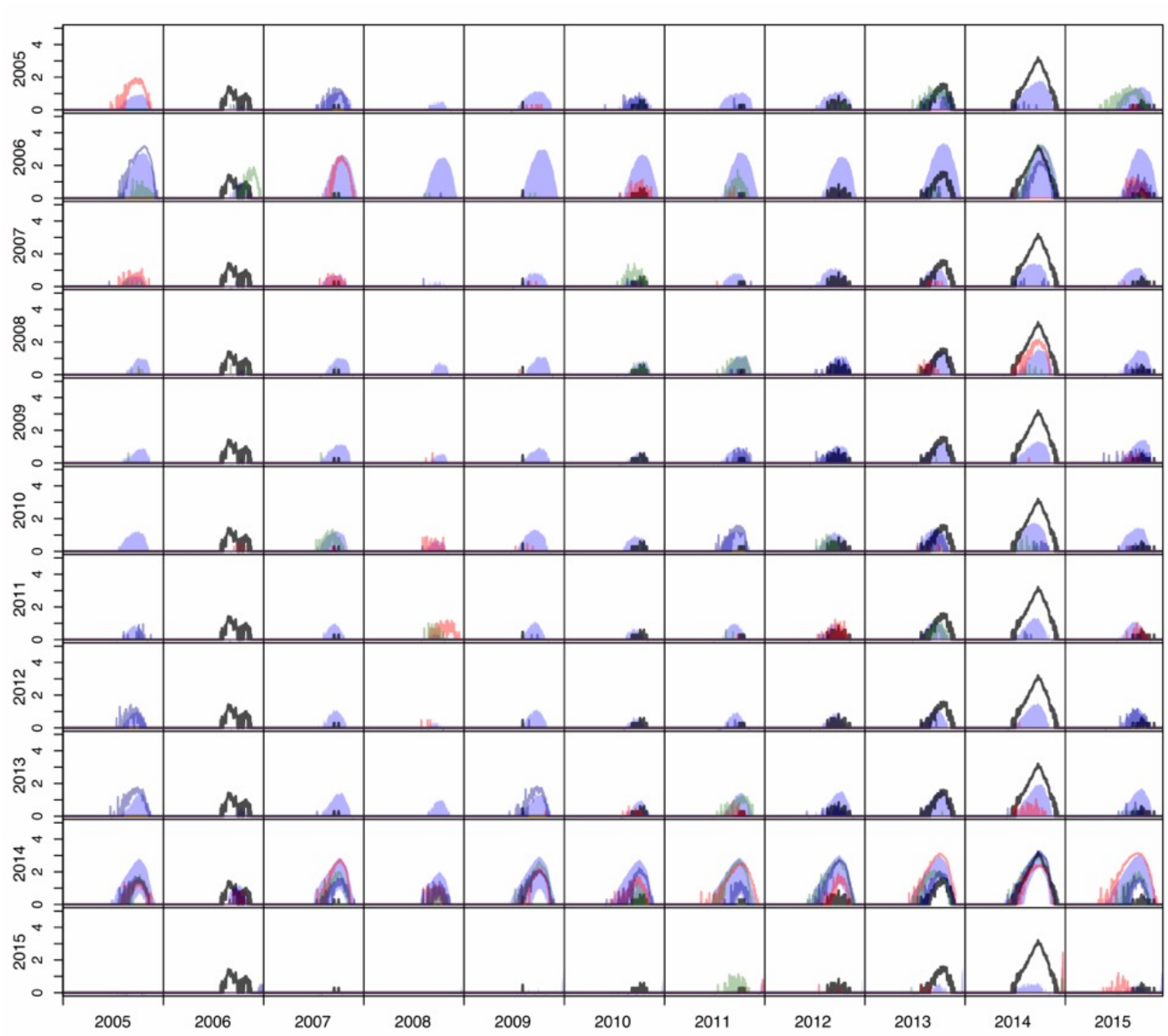

Supplementary Fig. 5. Epidemic trajectories (log_{10} scale) from the factorial experiment for each combination of local condition years (rows) and imported case years (columns). The shaded regions represent 95% posterior predictive intervals. Black lines represent observed values of daily local incidence. Colored lines show three different random samples from the epidemic trajectory posterior.

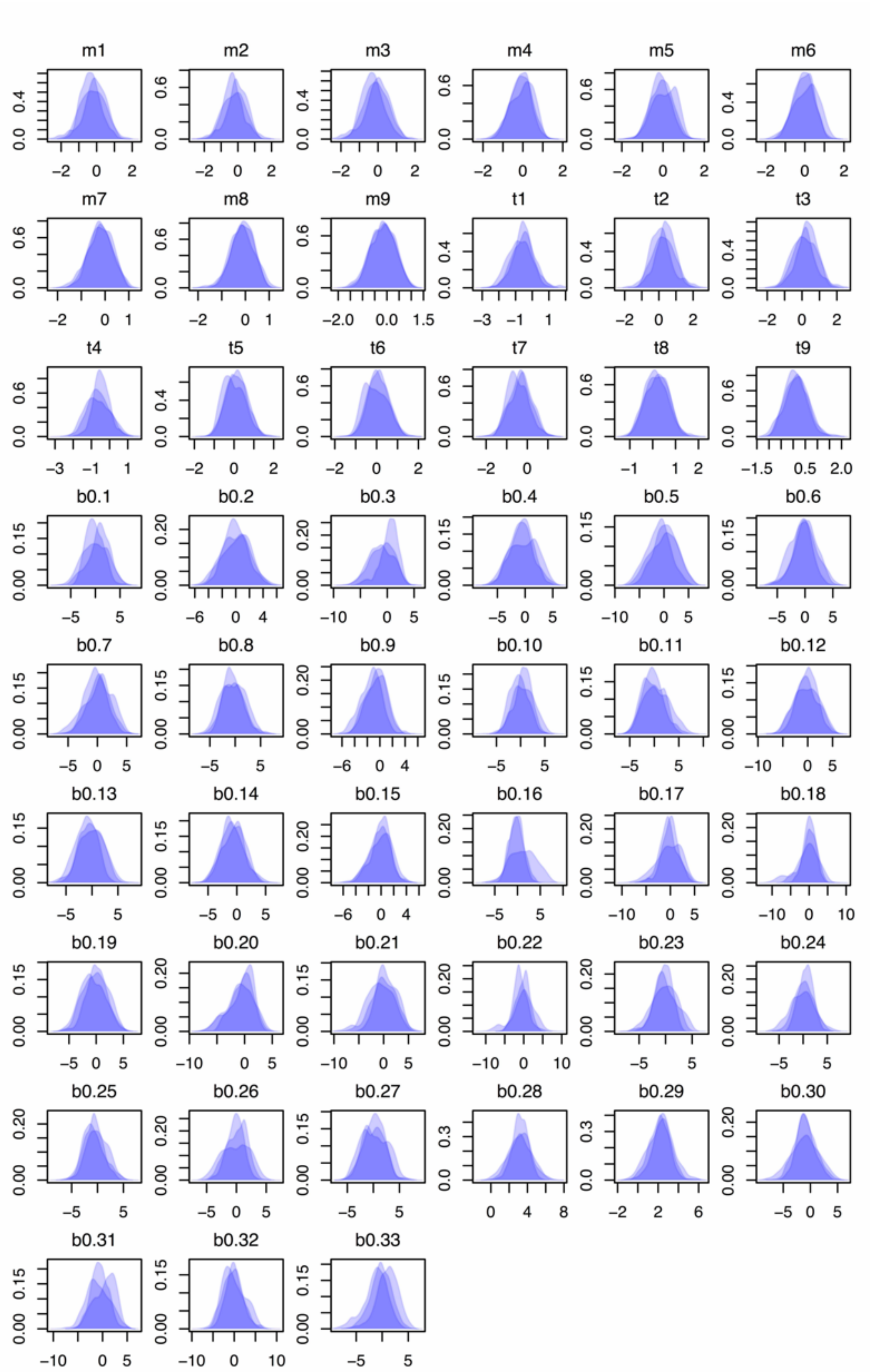

Supplementary Fig. 6. Posterior distributions for parameters estimated using Sequential Monte Carlo sampling. Within each plot, each shaded region represents the posterior distribution for a parameter from one independent SMC run.

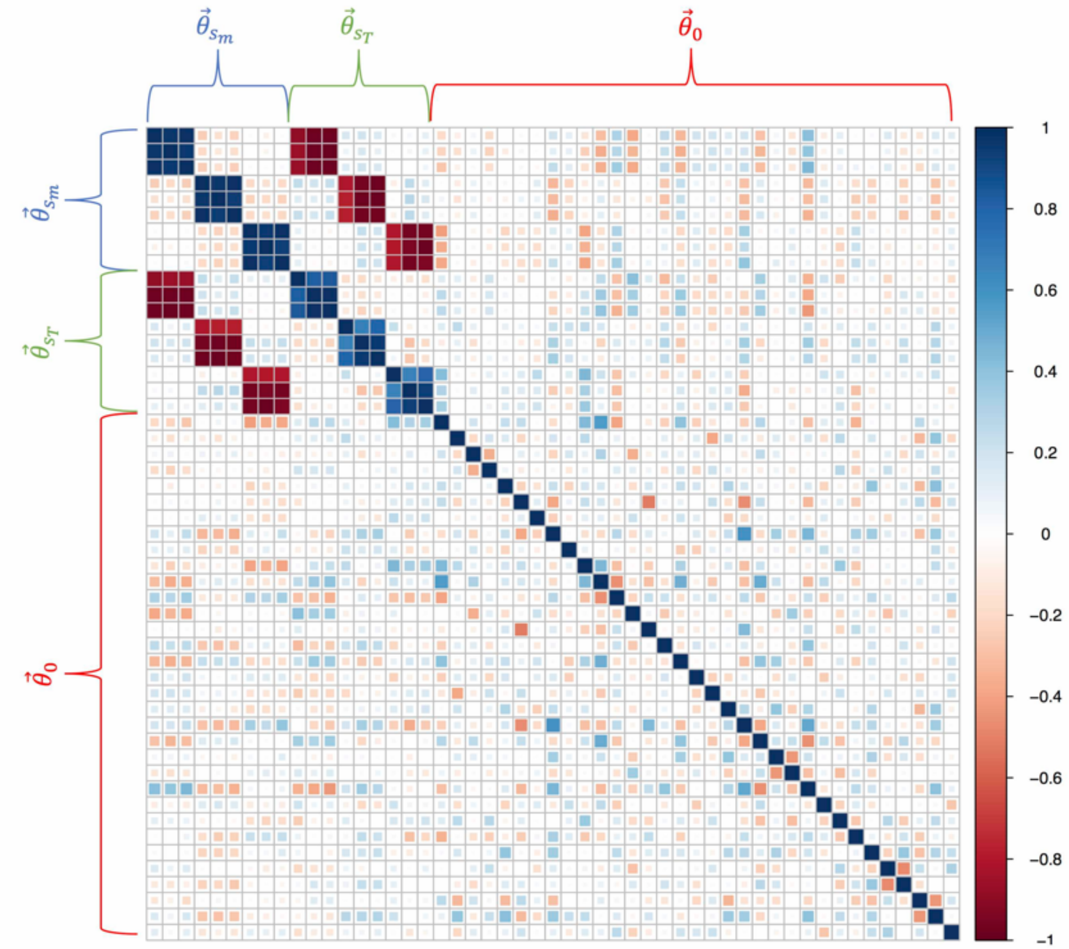

Supplementary Fig. 7. Correlation matrix for posterior estimates of parameters. Brackets indicate which entries in the matrix belong to which set of parameters. Dark blue corresponds to a large positive correlation and dark red corresponds to a large negative correlation.

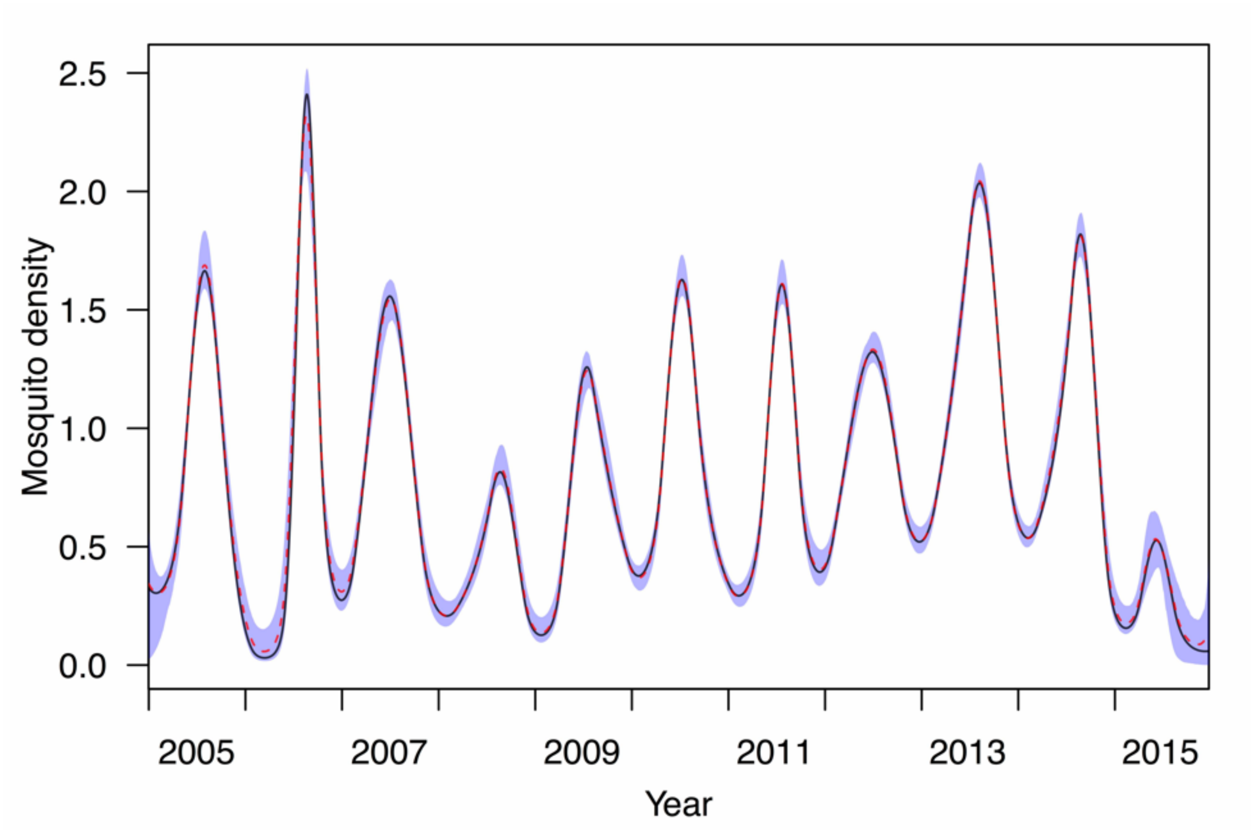

Supplementary Fig. 8. Confidence interval surrounding mosquito spline estimates. Blue shaded region is the 95% confidence interval. Red dotted line is the median mosquito spline estimate. Black line is the maximum likelihood estimate.

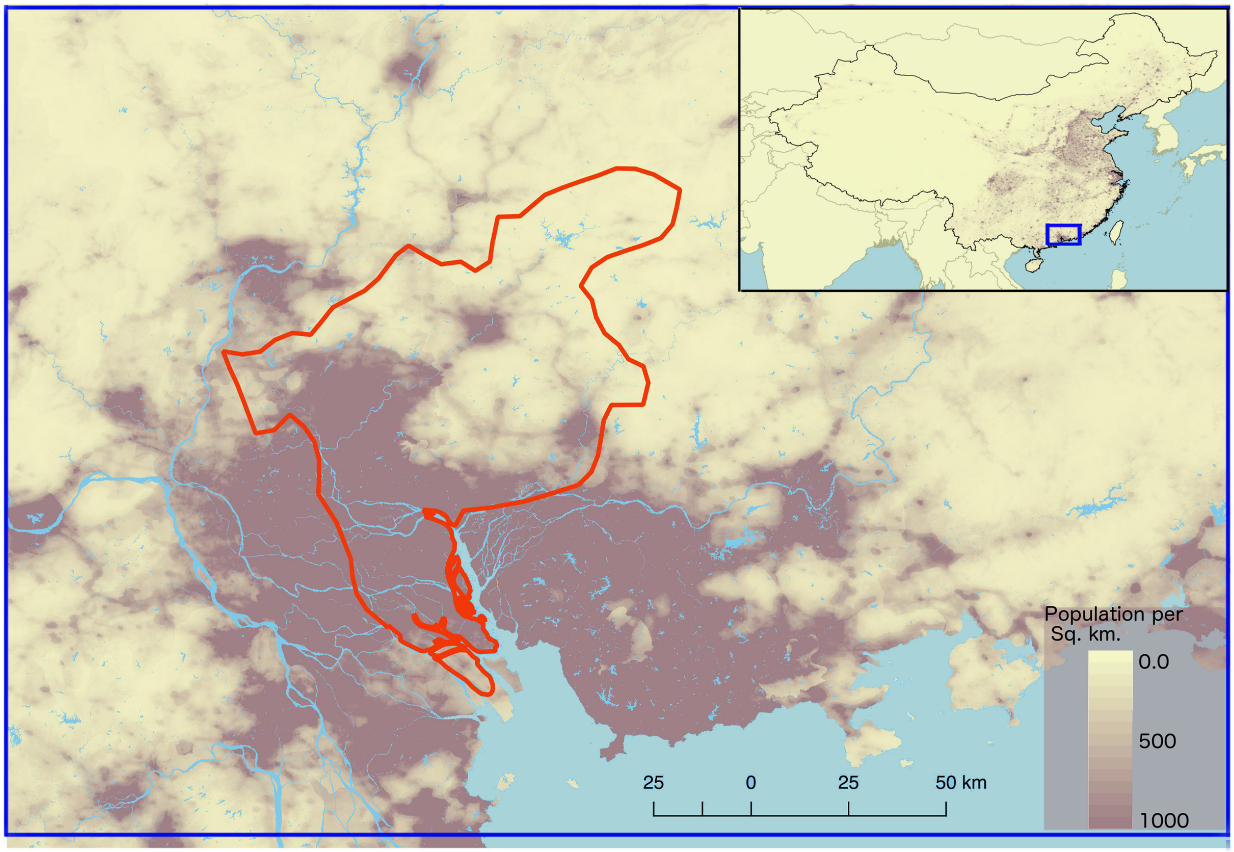

Supplementary Fig. 9. The city of Guangzhou in the province of Guangdong, China. The city limits of Guangzhou are outlined in red with population per square kilometer[8] for the region and the entirety of China (upper right)[9].

Supplementary Methods 1. Simpler alternative model.

In addition to the model we used throughout our analysis, we also considered a simpler alternative model to explore the extent to which our more complex model formulation was necessary to address our motivating questions about drivers of inter-annual variation in local incidence. As a prerequisite to addressing our motivating questions through factorial simulation experiments, it is essential that a model be capable of reproducing observed incidence patterns in Guangzhou during 2005-2015. If a model were to fail in this regard, then its predictions about incidence patterns under alternative conditions could not be viewed as reliable.

Model description

To formulate an alternative model with at least some key features of our primary model but that could be viewed as a simpler alternative, we followed the approach of Kraemer *et al.*[10]. Their model was also posed as a TSIR model and made use of the same effective number of cases in the previous generation, I'_t. Consistent with assumptions of our primary model, we assumed that transmission had a low impact on population susceptibility and that $I_t = \beta(t)\, I'_t$. The transmission coefficient $\beta(t)$ depended on a concave function of temperature, a monotonically increasing function of mosquito density, and a time-varying function to capture residual variation in transmission, similar to $\beta_0(t)$ in our primary model. These component functions were modeled with cubic smoothing splines using the s function in the scam package[11] in R, with concavity imposed with the bs= 'cv' option and monotonicity imposed with the bs= 'mpi' option (see model code below). These constraints are consistent with the prior assumptions applied to our primary model. One of the most distinct differences between the structure of this model and that of our primary model is that the former only made use of temperature and mosquito density values at time *t* rather than lagged over the 49 days preceding *t*.

```
model = scam(I_new ~ I_old + s(mean_temp, bs = 'cv') +
        s(mosq_spline, bs = 'mpi')+ s(time), family =
        'poisson', data = df)
```

Model fit

We used the scam package[11] in R to fit this model to data from days *t* for which both I_t and I'_t were positive (n=630). The scam model described a high proportion of the of variation in incidence, with the adjusted coefficient of variation, $R^2 = 0.955$. We then used this fitted model to simulate local dengue incidence over the entire timeframe of 4,017 days. Although the model described a high proportion of variation in the data to which it was fitted, the model-predicted incidence showed overall poor consistency with observed incidence patterns (Supplementary Methods 1 Fig. 1). For example, on the basis of Pearson's correlation coefficient between median values from model simulations and observed values, this model had a relatively low value of $\rho = 0.269$ compared to $\rho = 0.966$ for the more complex model used in our primary analysis. As a result, we conclude that use of the more complex model is indeed justified, as a relatively simple alternative model with similar features was unable to reproduce observed incidence patterns. It is likely that the most important reason that the more complex model displayed greater consistency with observed incidence patterns is that it was fitted specifically on the basis of its ability to reproduce those patterns rather than the simpler and more common alternative of being fitted on the basis of the model's ability to reproduce incidence patterns from one generation to the next. In particular, the fact that the simpler model did not account for days with zero incidence was likely a major limiting factor.

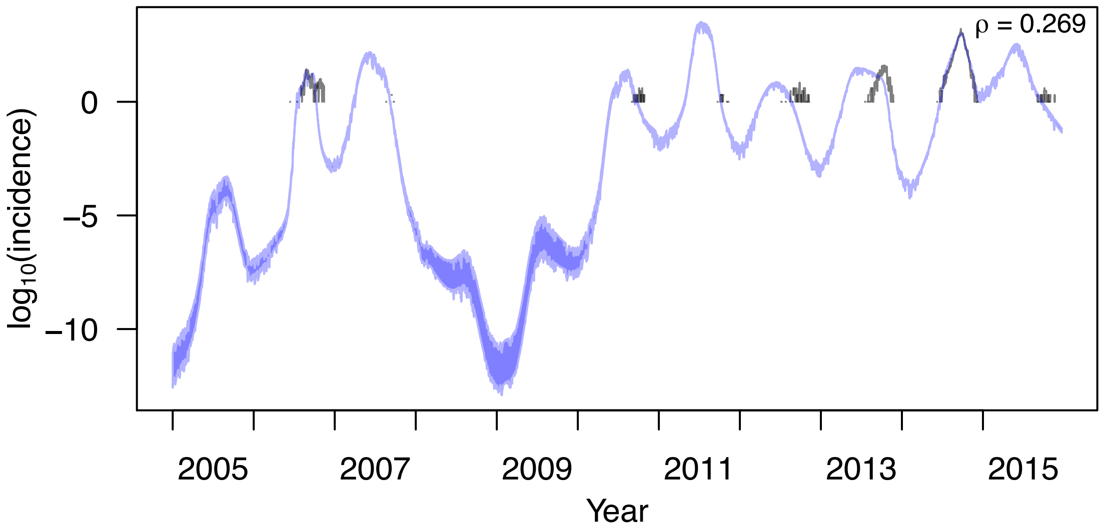

Supplementary Methods 1 Fig. 1. Correspondence between empirical and predicted patterns of local dengue incidence. Black line shows empirical values of log local incidence and the blue band shows the 95% confidence interval from the model fit. The value of Pearson's correlation coefficient indicated in the upper right pertains to untransformed daily values between model simulations and data.

Supplementary Methods 2. Alternative model formulations.

To consider the possibility that model formulations other than that used in our primary analysis might improve model performance, we fitted and performed validation analyses for several alternative models. These alternative models included other covariates in addition to the two explicit covariates used in our primary analysis (temperature and mosquito density) and a constant, rather than time-varying, residual term (β_0). Following the model used in our primary analysis, each alternative model was a variant of the TSIR model, $I_t = \beta(t)\frac{I'_t}{N}S'_t$, where the time-varying transmission coefficient, $\beta(t)$, is a function of lagged local conditions. For the model in our primary analysis that accounts for mosquito density, temperature, and other local conditions, $\beta(t) = f(m(t), T, \beta_0)$, meaning $\beta(t) = e^{\sum_{\tau=1}^{49} s_T(T_{t-\tau},\tau)}e^{\sum_{\tau=1}^{49} s_m(m(t-\tau),\tau)}e^{\beta_0(t)}$. The following alternative models are incorporated into $\beta(t)$ in a similar manner.

Alternative Model 1: $\beta(t) = f(m(t), T, \beta_0)$
This model excluded the time-varying residual term, $\beta_0(t)$, that we used in the model in our primary analysis. Doing so provides a direct examination of whether available covariates are, by themselves, capable of explaining inter-annual variation in dengue incidence. To implement this model, we included a constant residual term, β_0.

Alternative Model 2: $\beta(t) = f(m(t), T, P, \beta_0(t))$
The second alternative model we considered included precipitation as an additional covariate. We considered precipitation because standing water is required for juvenile mosquito stages[12]. The way in which precipitation affects DENV transmission is not always straightforward though. On the one hand, more precipitation can lead to higher transmission due to increased abundance of mosquitoes[12]. On the other hand, too much precipitation can flood standing water and flush eggs out of aquatic habitats[13]. Analogous to relationships between temperature and DENV transmission in the primary model, we specified an additional bivariate basis function for precipitation, $s_P(P_{t-\tau},\tau)$, to allow for $\beta(t)$ to depend on weighted sums of daily effects of temperature, $T_{t-\tau}$, mosquito abundance, $m(t-\tau)$, and precipitation, $P_{t-\tau}$. We used cubic B-splines to describe these functions using the fda package in R[14]. The precipitation covariate surface was defined by parameters associated with a 3x3 matrix that defined the height of each component of the bivariate spline ranging 1-49 days for τ and 0-215 mm for P.

Alternative Model 3: $\beta(t) = f(m(t), T, P, RH, \beta_0(t))$
The third alternative model we considered included both precipitation and relative humidity as additional covariates. Although precipitation and humidity may be somewhat collinear in their effects on mosquito abundance, as high relative humidity may be an indicator of rain, humidity has additionally been shown to independently affect adult mosquito mortality[15]. Again, analogous to relationships between temperature and DENV transmission in the primary model, we specified additional bivariate basis functions for precipitation, $s_P(P_{t-\tau},\tau)$, and relative humidity, $s_{RH}(RH_{t-\tau},\tau)$, to allow for $\beta(t)$ to depend on weighted sums of daily effects of temperature, $T_{t-\tau}$, mosquito abundance, $m(t-\tau)$, precipitation, $P_{t-\tau}$, and relative humidity, $RH_{t-\tau}$. Like other surfaces, the relative humidity covariate surface was defined with a bivariate spline ranging from 1-49 days for τ and 0-100% for RH.

Alternative Model 4: $\beta(t) = f(m(t), T, RH, \beta_0(t))$
The fourth alternative model we considered included only relative humidity as a covariate in addition to the primary model. As precipitation affects DENV transmission in complex ways, we constructed a model that would isolate the effects of relative humidity on transmission. Similar to the primary model and Alternative Models 2 and 3, we specified a bivariate basis function for

temperature, mosquito abundance, and relative humidity and allowed transmission to depend on the weighted sums of daily effects of these covariates.

Model fits

As with our primary model in the main text, we estimated the posterior distribution of parameters for Alternate Models 1-4 using a Sequential Monte Carlo algorithm implemented using the BayesianTools R library[16].

To validate the performance of each fitted model, we used data on imported cases to seed 1,000 simulations of local DENV transmission over the entire 2005-2015 time period. Over the period as a whole, Alternative Models 3 & 4—which included $\beta_0(t)$ and relative humidity as an additional covariate—had daily medians of simulated local dengue incidence that were highly correlated with observed local incidence, with $\rho = 0.962$ (Supplementary Methods 2 Fig. 5) and $\rho = 0.9122$ (Supplementary Methods 2 Fig. 4), respectively (Supplementary Methods 2 Table 1). Alternative Model 2—which only included precipitation as an additional covariate—had low correlation between simulated and observed local incidence ($\rho = 0.271$, Supplementary Methods 2 Fig. 2), while Alternative Model 1—which had no time-varying residual term—had essentially no correlation with observed local incidence ($\rho = -0.007$, Supplementary Methods 2 Fig. 1). In terms of their predictions about the relative probability of ranking different seasons high or low, none of Alternative Models 1-4 produced rankings as consistent with observed incidence patterns as the primary model (compare Supplementary Methods 2 Figs. 10-13 with Fig. 5). For the observed epidemiological "features" of local dengue incidence patterns, we found that each model performed similarly in terms of the number of years that the observed values fell within the 95% posterior predictive intervals (PPIs) (Supplementary Methods 2 Table 1). However, the range of the simulated values for the features differed greatly among models, due to differing levels of uncertainty in their predictions. For example, Alternative Model 1 had particularly wide ranges for each of the four epidemiological features (Supplementary Methods 2 Fig. 14), due to the lack of a time-varying residual term.

Given that the model used in our primary analysis showed strong support for a heightened contribution of residual variation from $\beta_0(t)$ in 2014, it was not surprising that Alternative Model 1 performed poorly. It was more surprising, however, that Alternative Models 2-4 all performed worse than our primary model, given that they did not exclude any of the predictors used in our primary model. In principle, a model that is nested within another (i.e., our primary model is a special case of Alternative Models 2-4) should do no better than a model that it is nested within, because additional parameters should only deviate from null values (i.e., parameter values resulting in the special case) if they lead to an improvement in the model's performance. On some level, the fact that Alternative Models 2-4 did not exceed the performance of the primary model suggests that they likely did not converge on their true global optimum values. Thus, we cannot exclude the possibility that other parameterizations of Alternative Models 2-4 could perform better than our primary model. In addition to model performance though, Alternative Models 2-4 exhibited some undesirable characteristics, including: 1) stronger dependence on $\beta_0(t)$ (Supplementary Methods 2 Figs. 7, 9); 2) erratic contributions from precipitation and relative humidity (Supplementary Methods 2 Figs. 7- 9); and 3) erratic patterns in $\beta(t)$. In addition, there were substantial gaps in the precipitation data (red bands in Supplementary Methods 2 Fig. 1), which likely exacerbated these problems for Alternative Models 2 & 3. Most importantly, none of these results indicate potential to provide a better explanation for inter-annual variation in dengue incidence, which was the primary goal of our analysis.

As a result of these assessments of Alternative Models 1-4, we concluded that our primary model—which includes $\beta_0(t)$, temperature, and mosquito density as covariates—was an

appropriate choice for modeling inter-annual variation in dengue incidence in our study area. Although precipitation and humidity are meaningful biologically[12,13], we suspect that much of the influence of those variables may be mediated through their effects on mosquito density, which was modeled directly in our primary model and may not be easily improved on by adding data on precipitation and humidity.

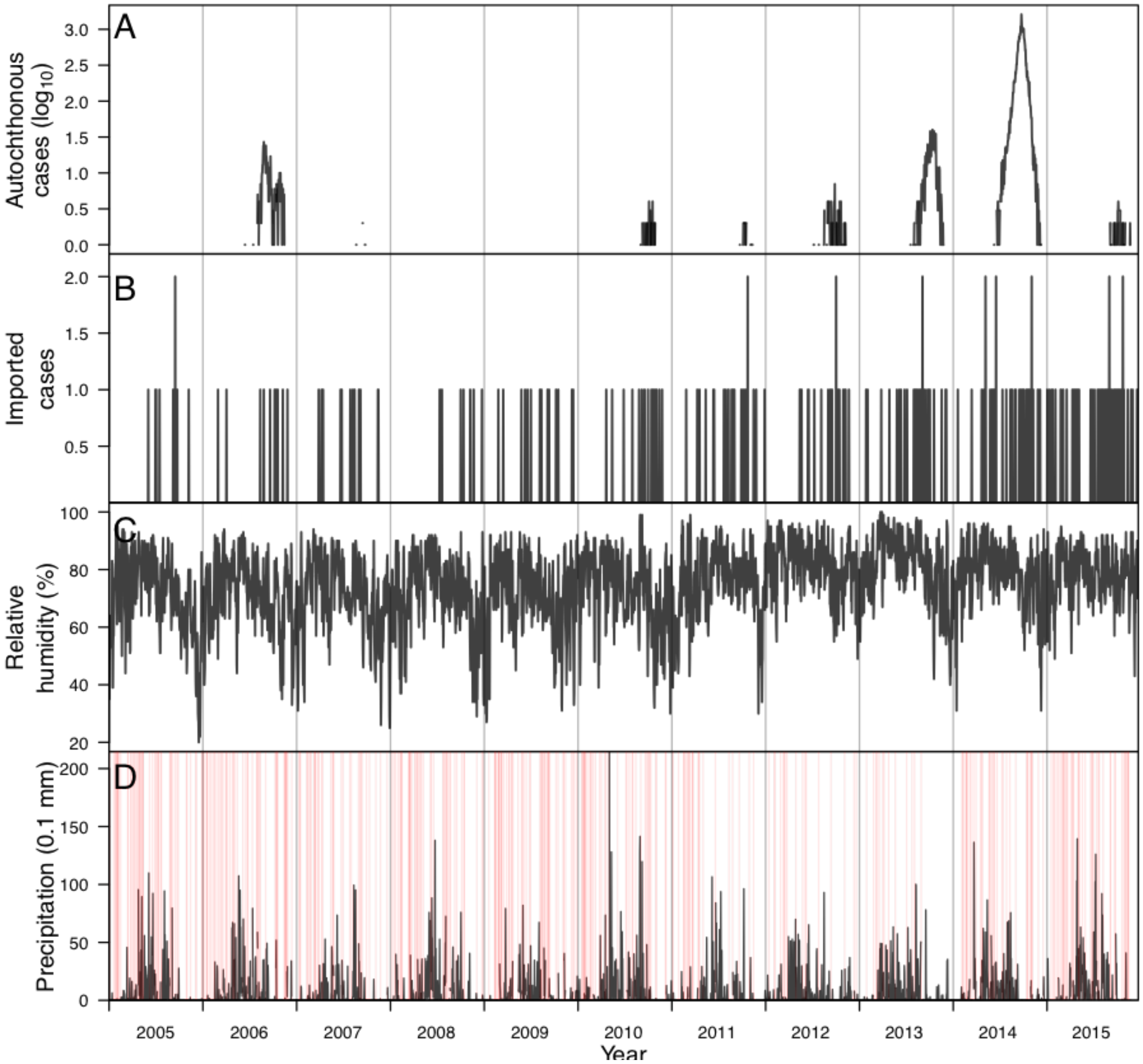

Supplementary Methods 2 Fig. 1. Time series of data from 2005-2015 in Guangzhou, China. (A) Local dengue incidence. (B) Imported dengue incidence. (C) Daily relative humidity. (D) Daily precipitation with red lines indicating days with missing data (e.g., snow, sleet, fog).

	**Primary model**	**Alt. Model 1**	**Alt. Model 2**	**Alt. Model 3**	**Alt. Model 4**
**Variables included**	$m(t) + T + \beta_0(t)$	$m(t) + T + \beta_0$	$m(t) + T + P + \beta_0(t)$	$m(t) + T + P + RH + \beta_0(t)$	$m(t) + T + RH + \beta_0(t)$
**Total dengue incidence (PPI)**	11/11	9/11	7/11	10/11	9/11
**Peak weekly incidence (PPI)**	11/11	10/11	10/11	10/11	9/11
**Number of weeks with non-zero dengue incidence (PPI)**	8/11	11/11	7/11	9/11	8/11
**Length of transmission season (PPI)**	8/11	11/11	6/11	8/11	5/11
$\boldsymbol{\rho}$	0.967	-0.007	0.271	0.9122	0.962

Supplementary Methods 2 Table 1. Model validation for alternative model formulations. The first four rows of results show the number of years that the Bayesian p-value fell between 0.025 and 0.975, the 95% posterior predictive interval (PPI), for the given epidemic feature. The last row is the Pearson's correlation coefficient (ρ) comparing the untransformed daily value of the median simulated patterns with empirical patterns of local dengue incidence. Graphical representations of the numerical values of the observed data and the 95% PPI for each model are shown in Figs. ST2.14-ST2.18.

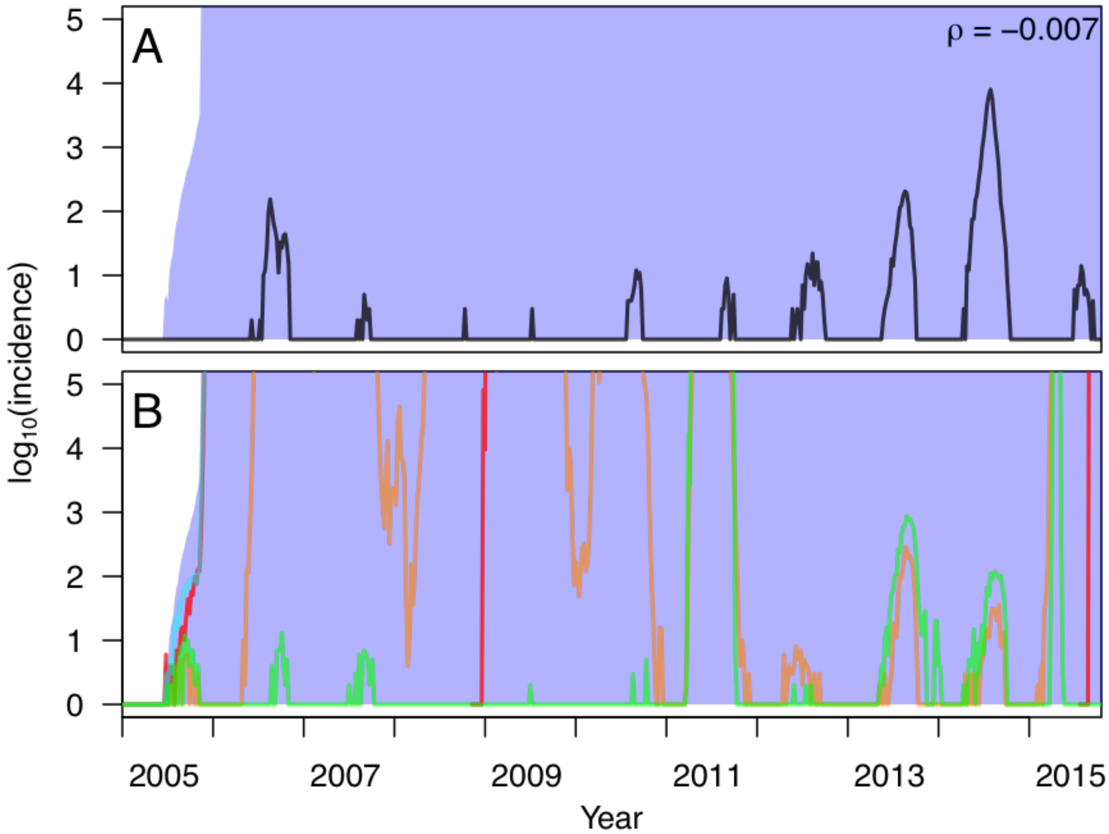

Supplementary Methods 2 Fig. 2. Correspondence between empirical and simulated patterns of local dengue incidence using a model in which the transmission coefficient was fitted to $m(t) + T + \beta_0$ (Alternative Model 1). In A, the black line shows empirical values of log local incidence and the blue band shows the 95% posterior predictive interval from model simulations. The value of Pearson's correlation coefficient indicated in the upper right pertains to untransformed daily values between model simulations and data. In B, different colored lines correspond to simulations based on different samples from the posterior distribution of parameter values. Simulations of local transmission were seeded by data on imported cases and otherwise used the fitted model of local transmission to simulate local cases.

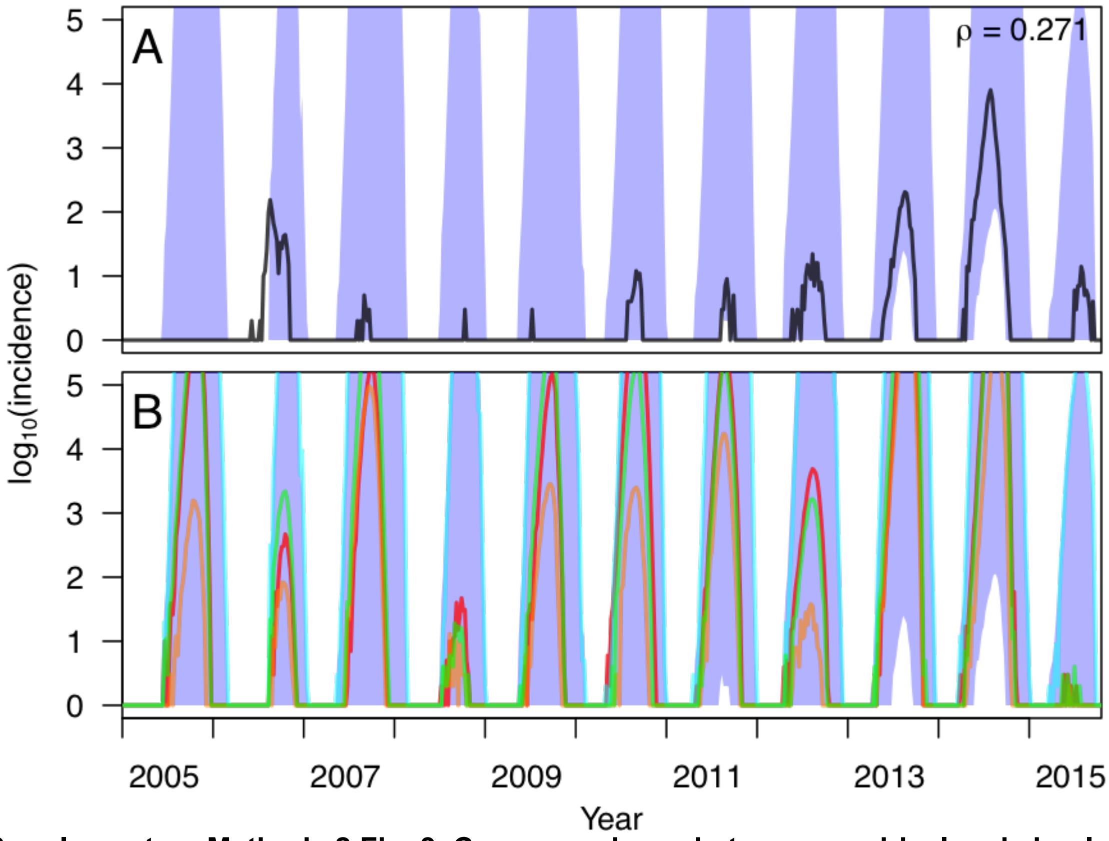

Supplementary Methods 2 Fig. 3. Correspondence between empirical and simulated patterns of local dengue incidence using a model in which the transmission coefficient was fitted to $m(t) + T + P + \beta_0(t)$ (Alternative Model 2). In A, the black line shows empirical values of log local incidence and the blue band shows the 95% posterior predictive interval from model simulations. The value of Pearson's correlation coefficient indicated in the upper right pertains to untransformed daily values between model simulations and data. In B, different colored lines correspond to simulations based on different samples from the posterior distribution of parameter values. Simulations of local transmission were seeded by data on imported cases and otherwise used the fitted model of local transmission to simulate local cases.

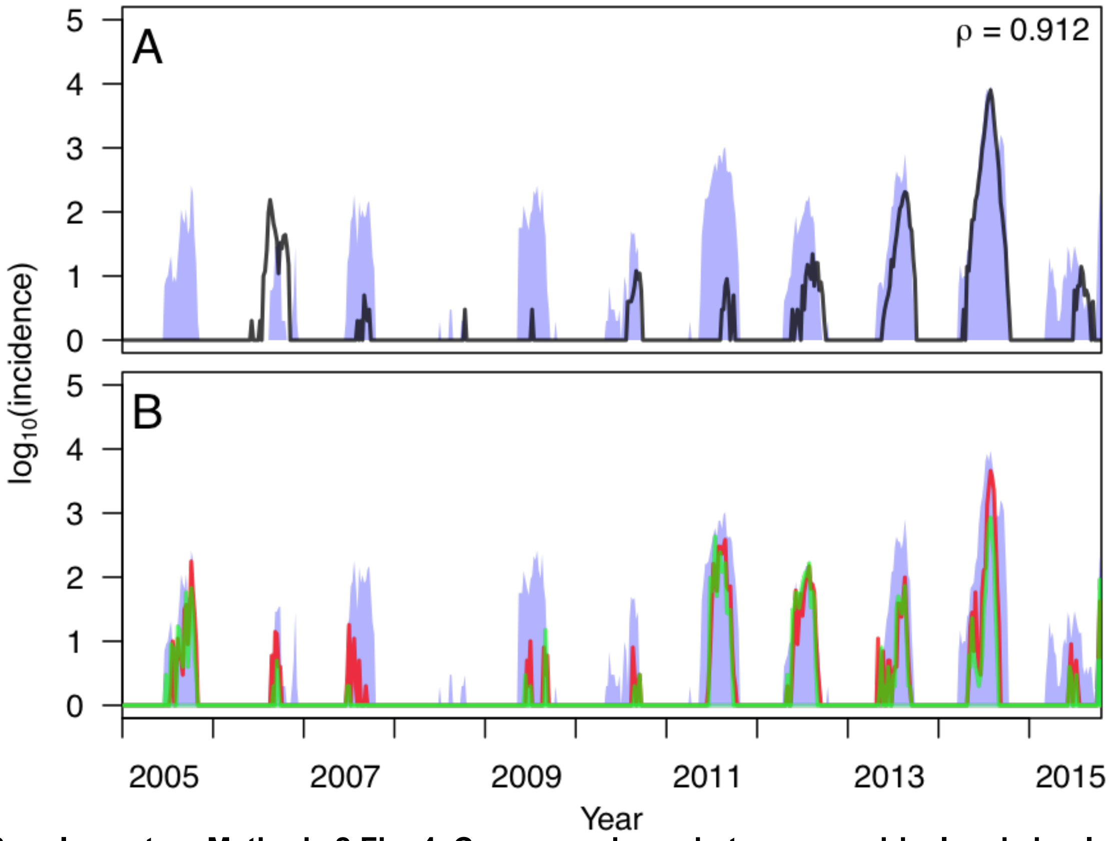

Supplementary Methods 2 Fig. 4. Correspondence between empirical and simulated patterns of local dengue incidence using a model in which the transmission coefficient was fitted to $\boldsymbol{m(t) + T + P + RH + \beta_0(t)}$ (Alternative Model 3). In A, the black line shows empirical values of log local incidence and the blue band shows the 95% posterior predictive interval from model simulations. The value of Pearson's correlation coefficient indicated in the upper right pertains to untransformed daily values between model simulations and data. In B, different colored lines correspond to simulations based on different samples from the posterior distribution of parameter values. Simulations of local transmission were seeded by data on imported cases and otherwise used the fitted model of local transmission to simulate local cases.

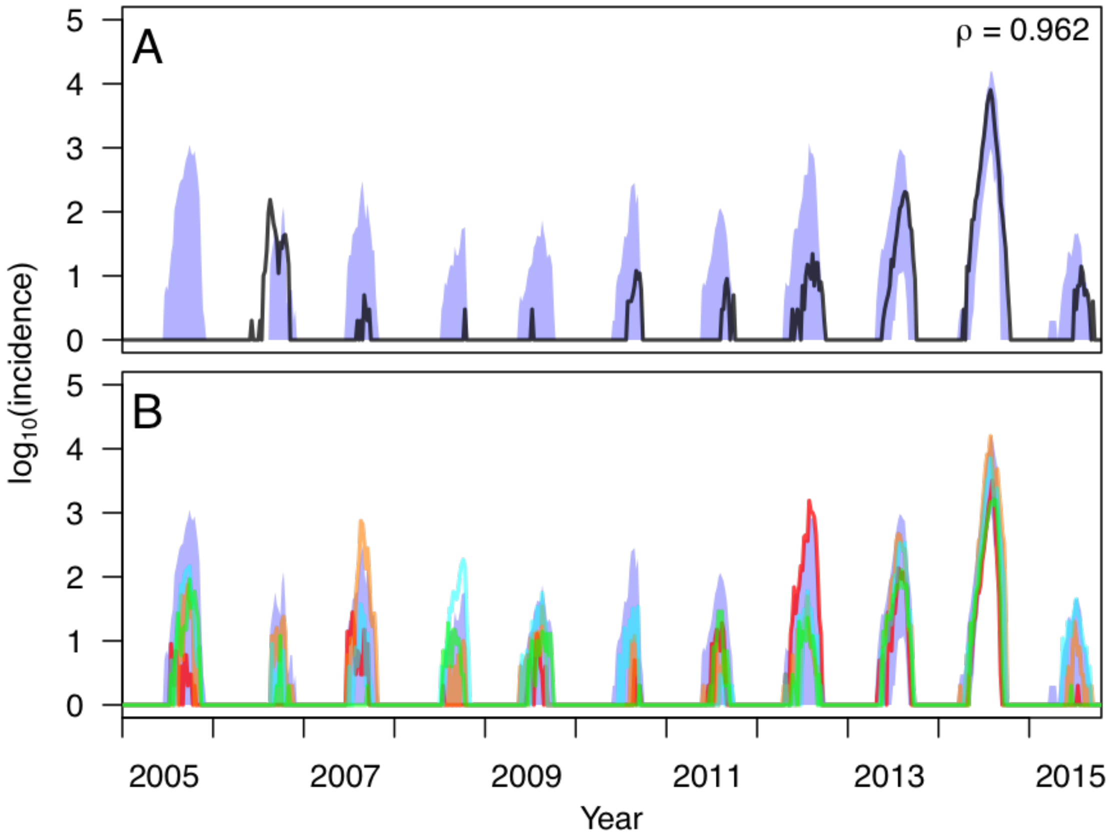

Supplementary Methods 2 Fig. 5. Correspondence between empirical and simulated patterns of local dengue incidence using a model in which the transmission coefficient was fitted to $\boldsymbol{m(t) + T + RH + \beta_0(t)}$ (Alternative Model 4). In A, the black line shows empirical values of log local incidence and the blue band shows the 95% posterior predictive interval from model simulations. The value of Pearson's correlation coefficient indicated in the upper right pertains to untransformed daily values between model simulations and data. In B, different colored lines correspond to simulations based on different samples from the posterior distribution of parameter values. Simulations of local transmission were seeded by data on imported cases and otherwise used the fitted model of local transmission to simulate local cases.

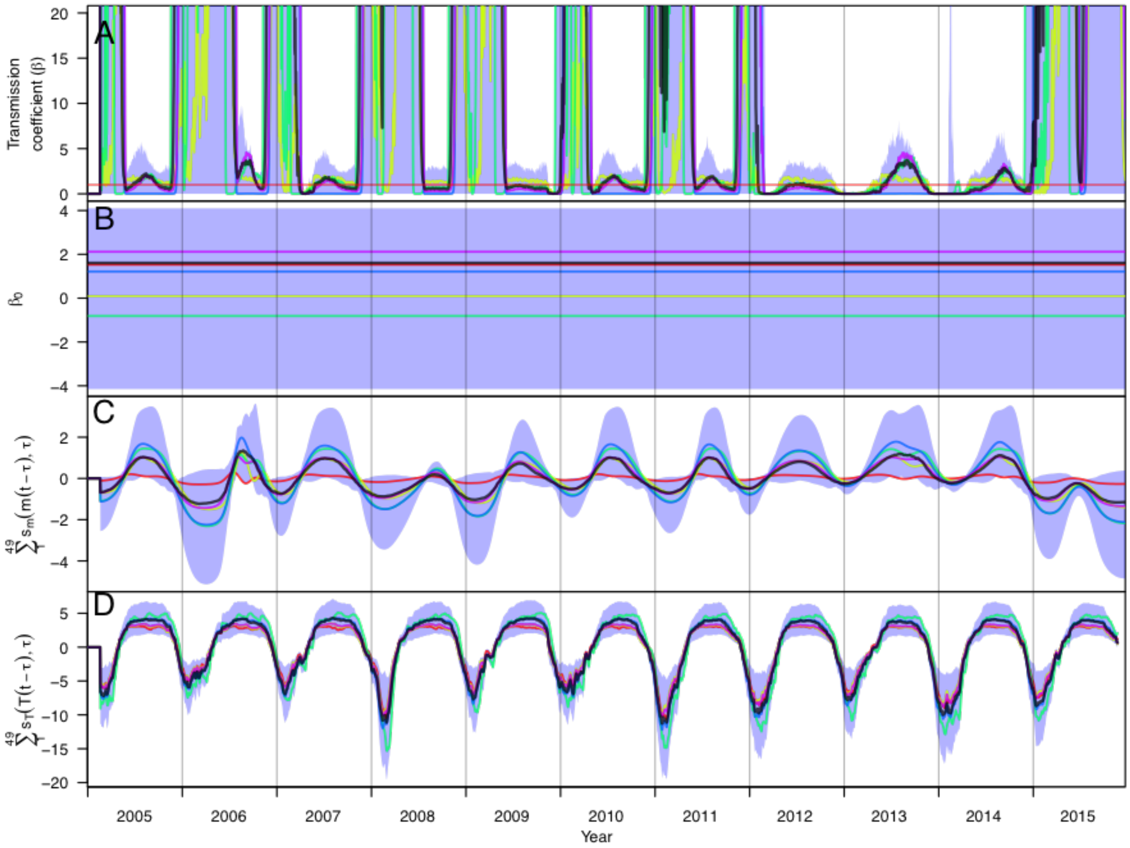

Supplementary Methods 2 Fig. 6. Time series of posterior estimates of the (A) time-varying transmission coefficient, $\beta(t)$, and contributions thereto from (B) constant residual local conditions, β_0, (C) mosquito density, $m(t)$, and (D) temperature for Alternative Model 1. Different colored lines correspond to different samples from the posterior distribution of parameter values, which provide information about correlations among parameters that pertain to different components of the model. The red horizontal line in A indicated $\beta(t) = 1$. The shaded blue region represents the 95% posterior predictive interval, and the black line is the median value.

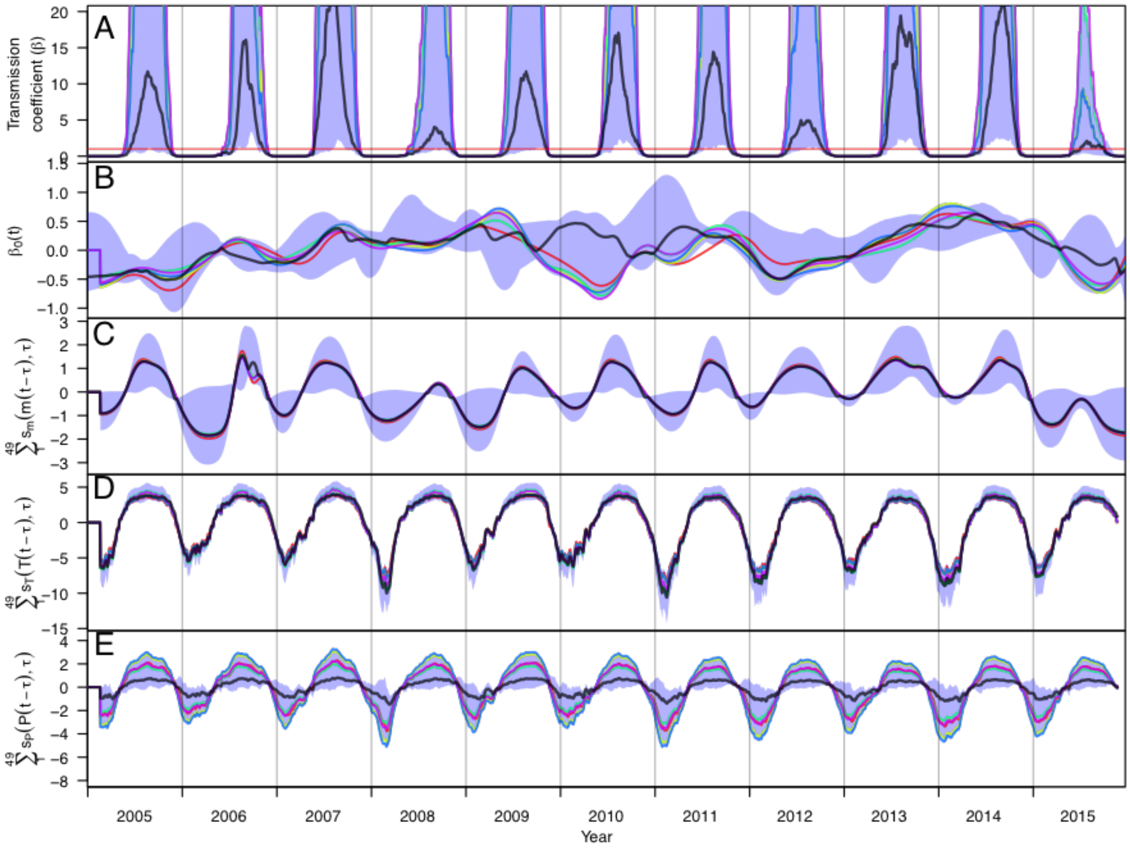

Supplementary Methods 2 Fig. 7. Time series of posterior estimates of the (A) time-varying transmission coefficient, $\boldsymbol{\beta(t)}$, and contributions thereto from (B) residual local conditions, $\boldsymbol{\beta_0(t)}$, (C) mosquito density, $\boldsymbol{m(t)}$, (D) temperature, and (E) precipitation for Alternative Model 2. Different colored lines correspond to different samples from the posterior distribution of parameter values, which provide information about correlations among parameters that pertain to different components of the model. The red horizontal line in A indicated $\beta(t) = 1$. The shaded blue region represents the 95% posterior predictive interval, and the black line is the median value.

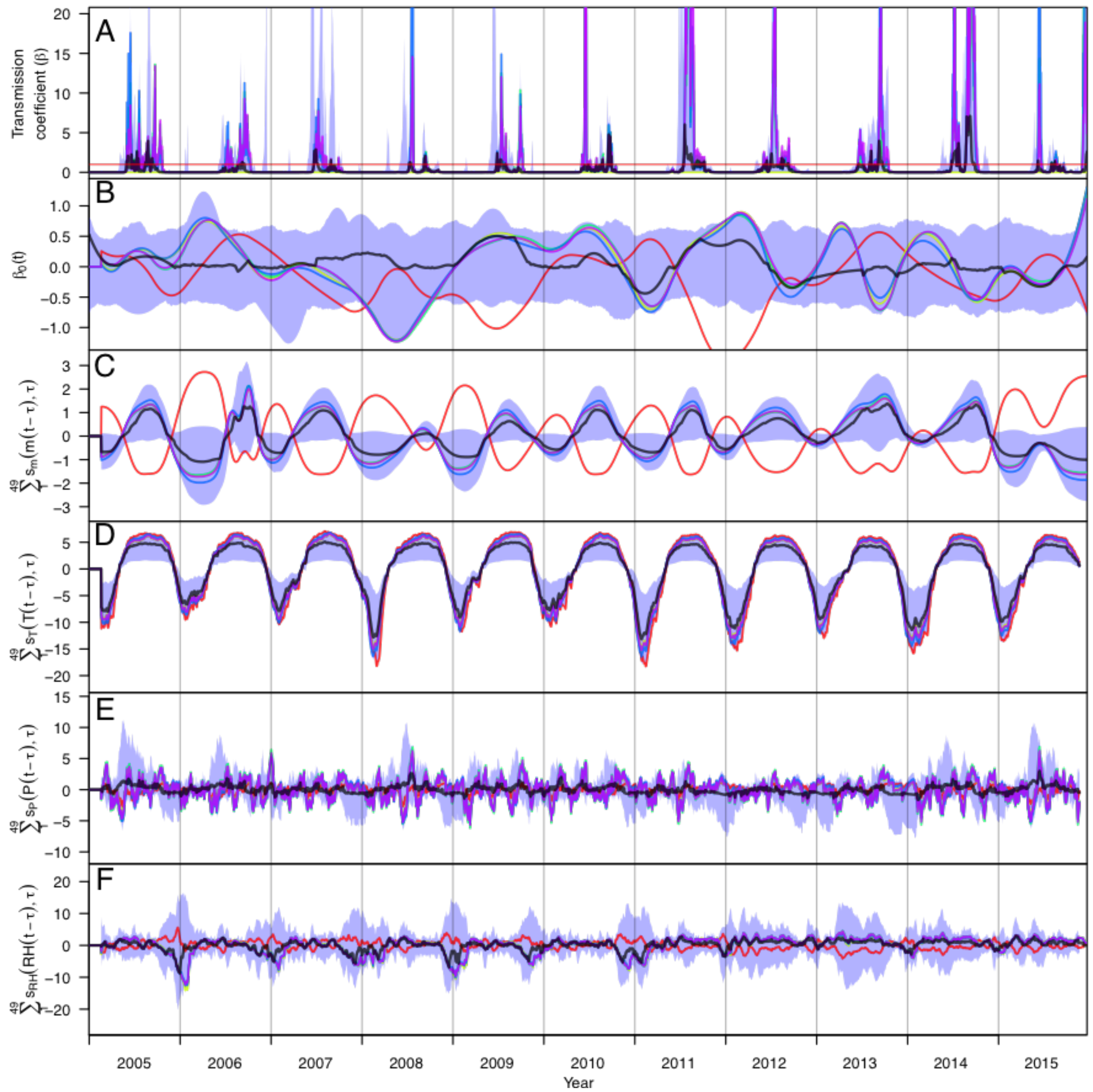

Supplementary Methods 2 Fig. 8. Time series of posterior estimates of the (A) time-varying transmission coefficient, $\beta(t)$, and contributions thereto from (B) residual local conditions, $\beta_0(t)$, (C) mosquito density, $m(t)$, (D) temperature, (E) precipitation, and (F) relative humidity for Alternative Model 3. Different colored lines correspond to different samples from the posterior distribution of parameter values, which provide information about correlations among parameters that pertain to different components of the model. The red horizontal line in A indicated $\beta(t) = 1$. The shaded blue region represents the 95% posterior predictive interval, and the black line is the median value.

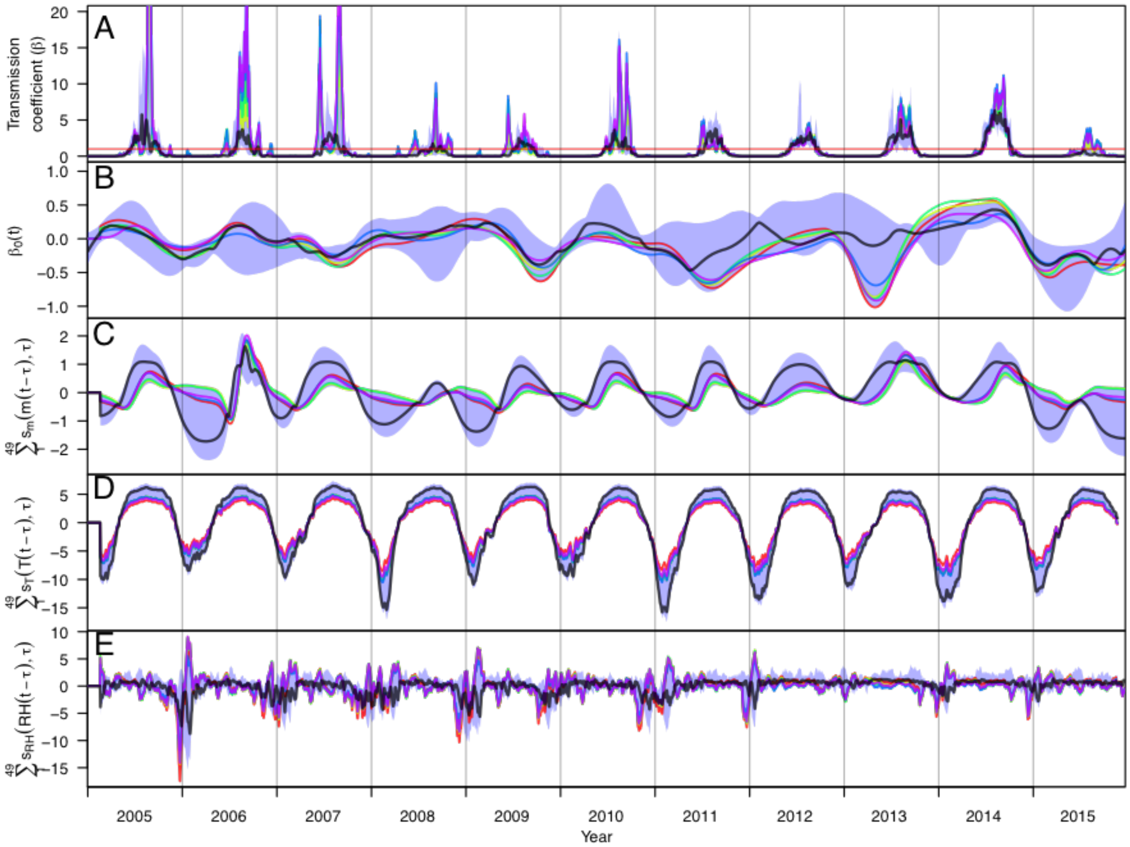

Supplementary Methods 2 Fig. 9. Time series of posterior estimates of the (A) time-varying transmission coefficient, $\boldsymbol{\beta(t)}$, and contributions thereto from (B) residual local conditions, $\boldsymbol{\beta_0(t)}$, (C) mosquito density, $\boldsymbol{m(t)}$, (D) temperature, and (E) relative humidity for Alternative Model 4. Different colored lines correspond to different samples from the posterior distribution of parameter values, which provide information about correlations among parameters that pertain to different components of the model. The red horizontal line in A indicated $\beta(t) = 1$. The shaded blue region represents the 95% posterior predictive interval, and the black line is the median value.

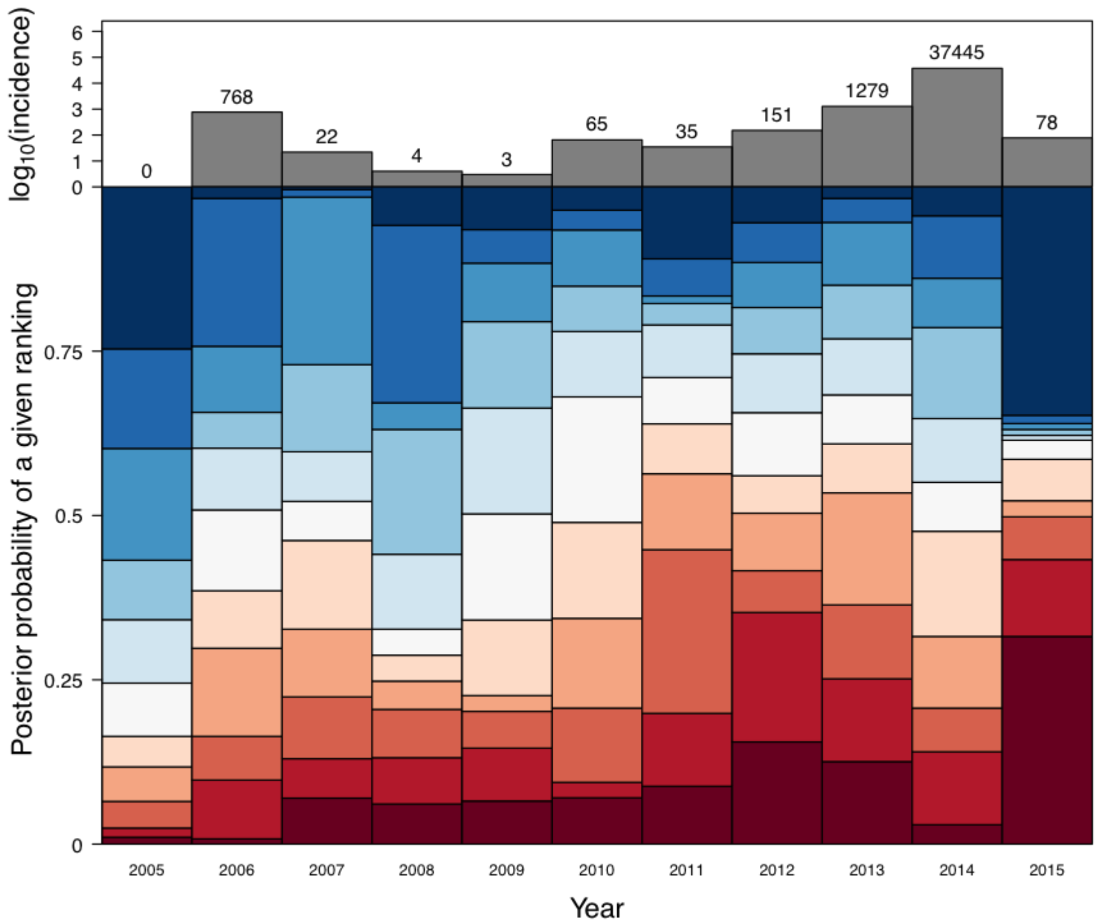

Supplementary Methods 2 Fig. 10. Ranking of years by annual local incidence simulated with the transmission coefficient was fitted to $m(t) + T + \beta_0$ given data on local conditions and imported cases from that year (Alternative Model 1). Dark red corresponds to the lowest ranking (i.e., highest simulated local incidence), and dark blue corresponds to the highest ranking (i.e., lowest simulated local incidence). The height of a given segment of a given year's bar is proportional to the posterior probability that simulations of local incidence from that year were of a given rank relative to other years.

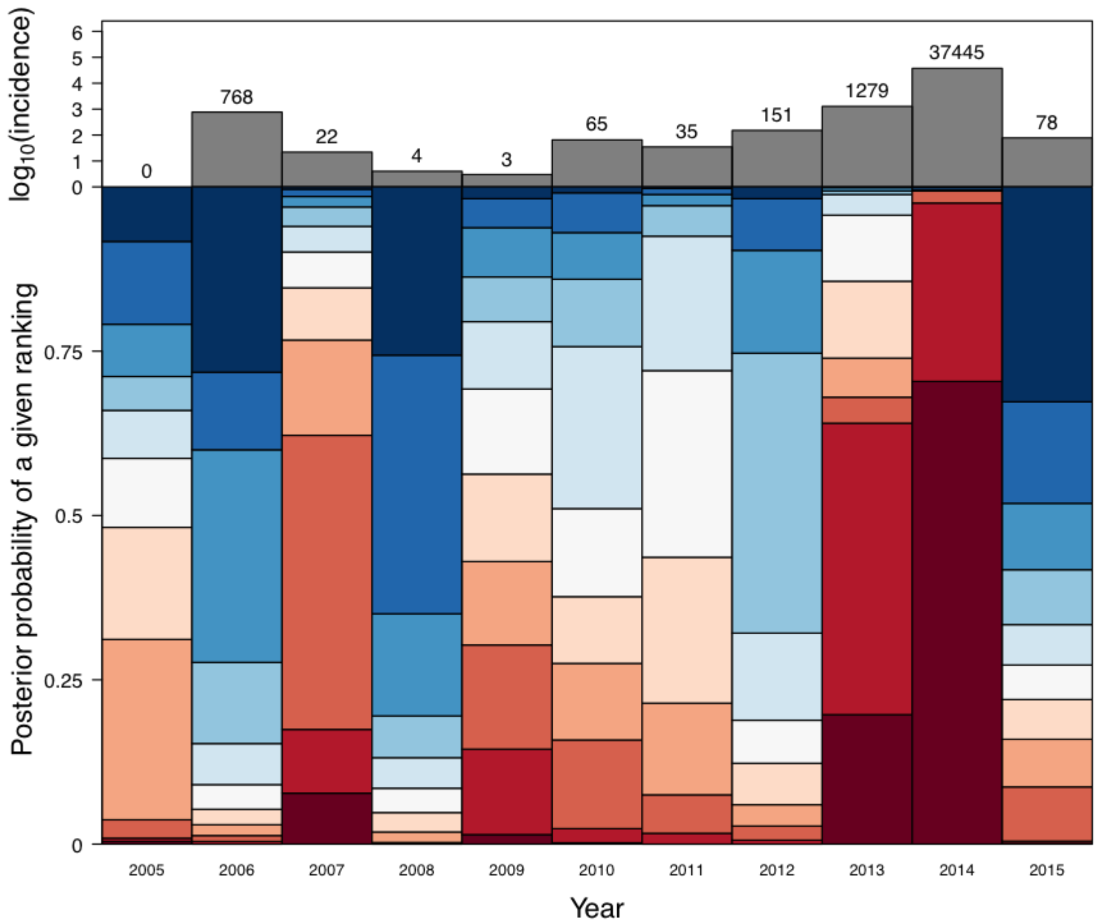

Supplementary Methods 2 Fig. 11. Ranking of years by annual local incidence simulated with the transmission coefficient was fitted to $m(t) + T + P + \beta_0(t)$ given data on local conditions and imported cases from that year (Alternative Model 2). Dark red corresponds to the lowest ranking (i.e., highest simulated local incidence), and dark blue corresponds to the highest ranking (i.e., lowest simulated local incidence). The height of a given segment of a given year's bar is proportional to the posterior probability that simulations of local incidence from that year were of a given rank relative to other years.

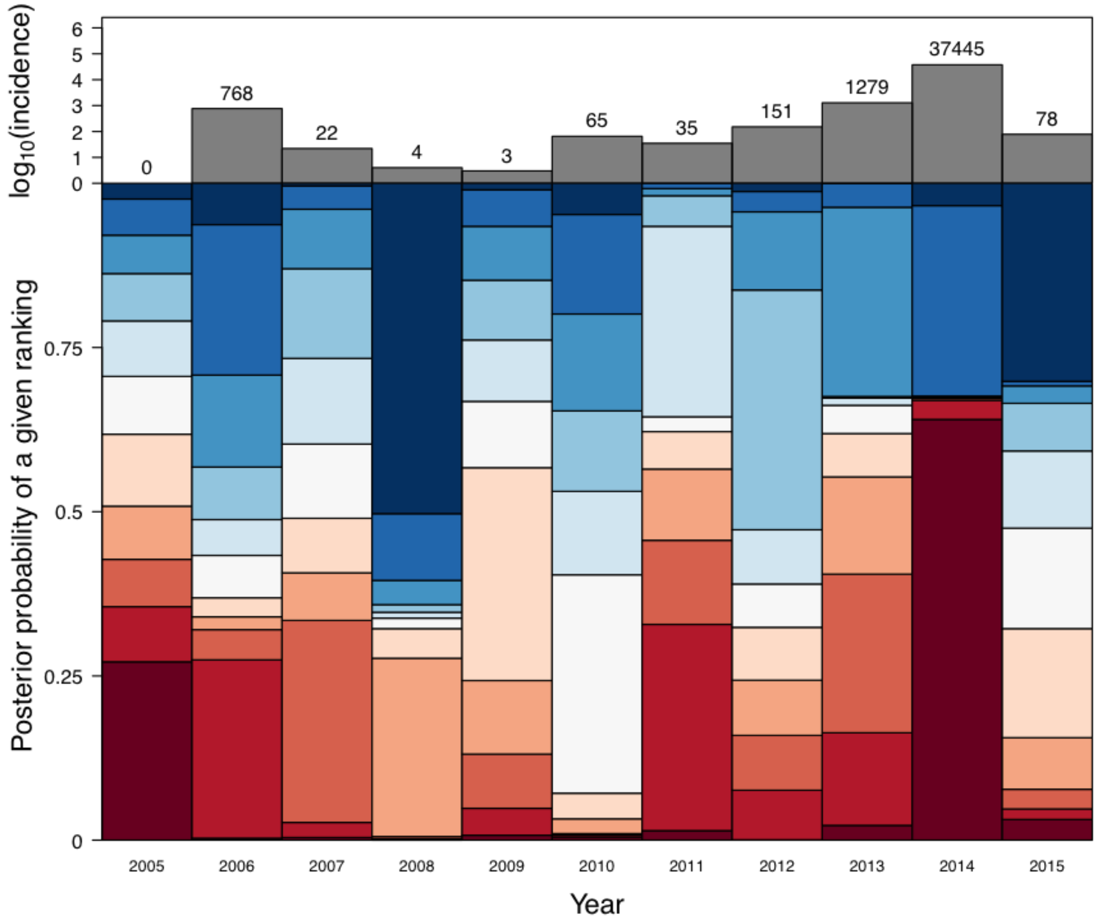

Supplementary Methods 2 Fig. 12. Ranking of years by annual local incidence simulated with the transmission coefficient was fitted to $m(t) + T + P + RH + \beta_0(t)$ given data on local conditions and imported cases from that year (Alternative Model 3). Dark red corresponds to the lowest ranking (i.e., highest simulated local incidence), and dark blue corresponds to the highest ranking (i.e., lowest simulated local incidence). The height of a given segment of a given year's bar is proportional to the posterior probability that simulations of local incidence from that year were of a given rank relative to other years.

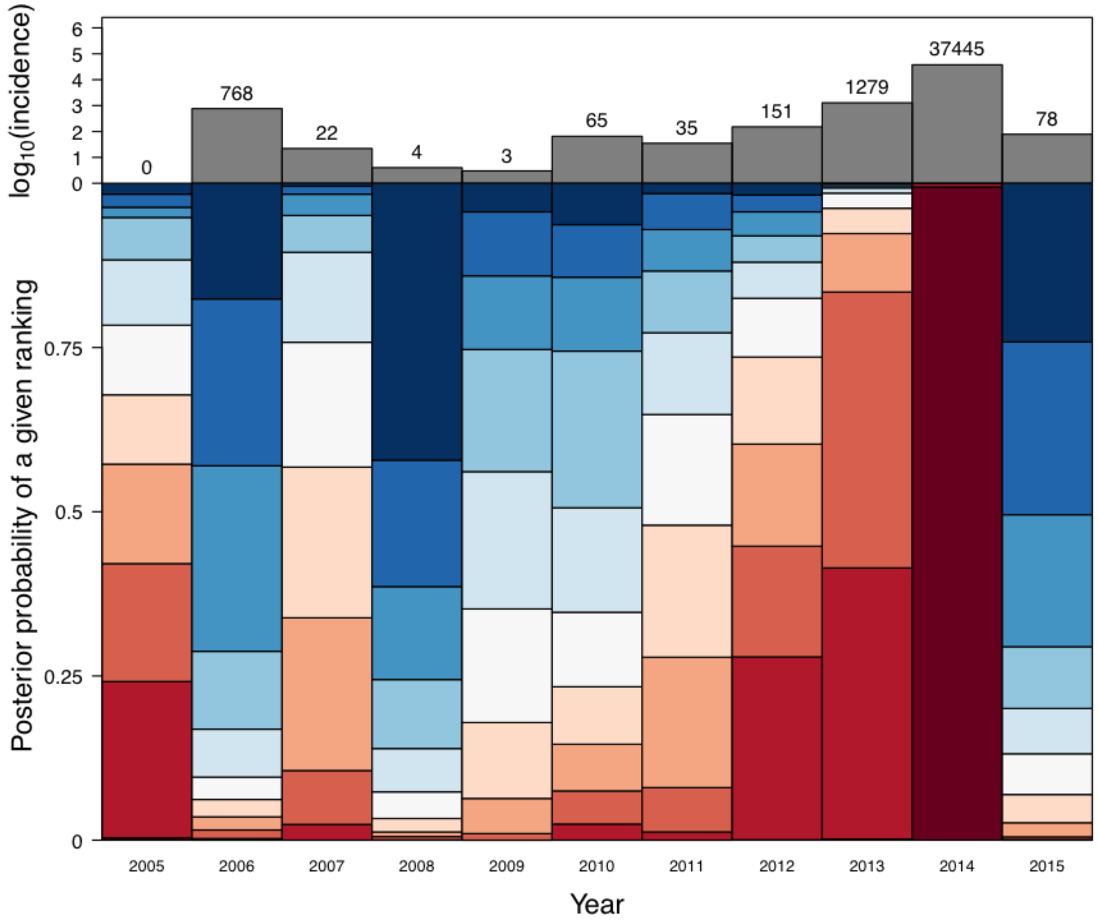

Supplementary Methods 2 Fig. 13. Ranking of years by annual local incidence simulated with the transmission coefficient was fitted to $m(t) + T + RH + \beta_0(t)$ given data on local conditions and imported cases from that year (Alternative Model 4). Dark red corresponds to the lowest ranking (i.e., highest simulated local incidence), and dark blue corresponds to the highest ranking (i.e., lowest simulated local incidence). The height of a given segment of a given year's bar is proportional to the posterior probability that simulations of local incidence from that year were of a given rank relative to other years.

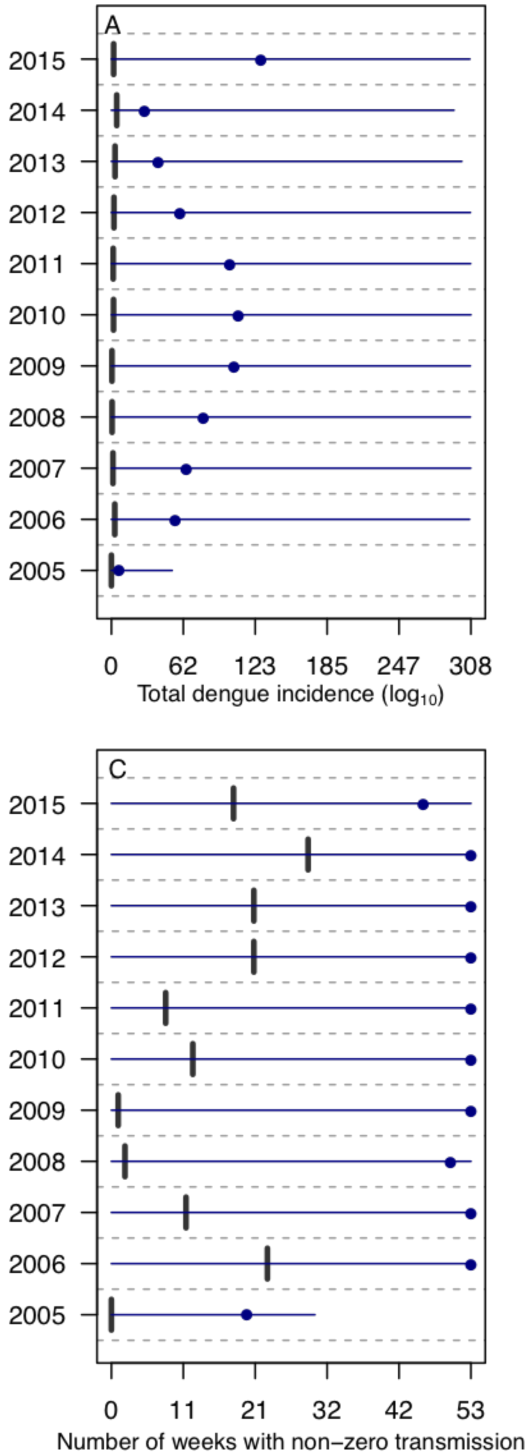

Supplementary Methods 2 Fig. 14. Median and 95% quantile for model simulations and empirical value for epidemiological characteristics for model with the transmission coefficient was fitted to $m(t) + T + \beta_0$ (Alternative Model 1). Points are median value, horizontal lines are 95% posterior quantile, and vertical line is empirical value for (A) total dengue incidence, (B) peak weekly incidence, (C) number of weeks with non-zero dengue incidence, and (D) length of transmission season.

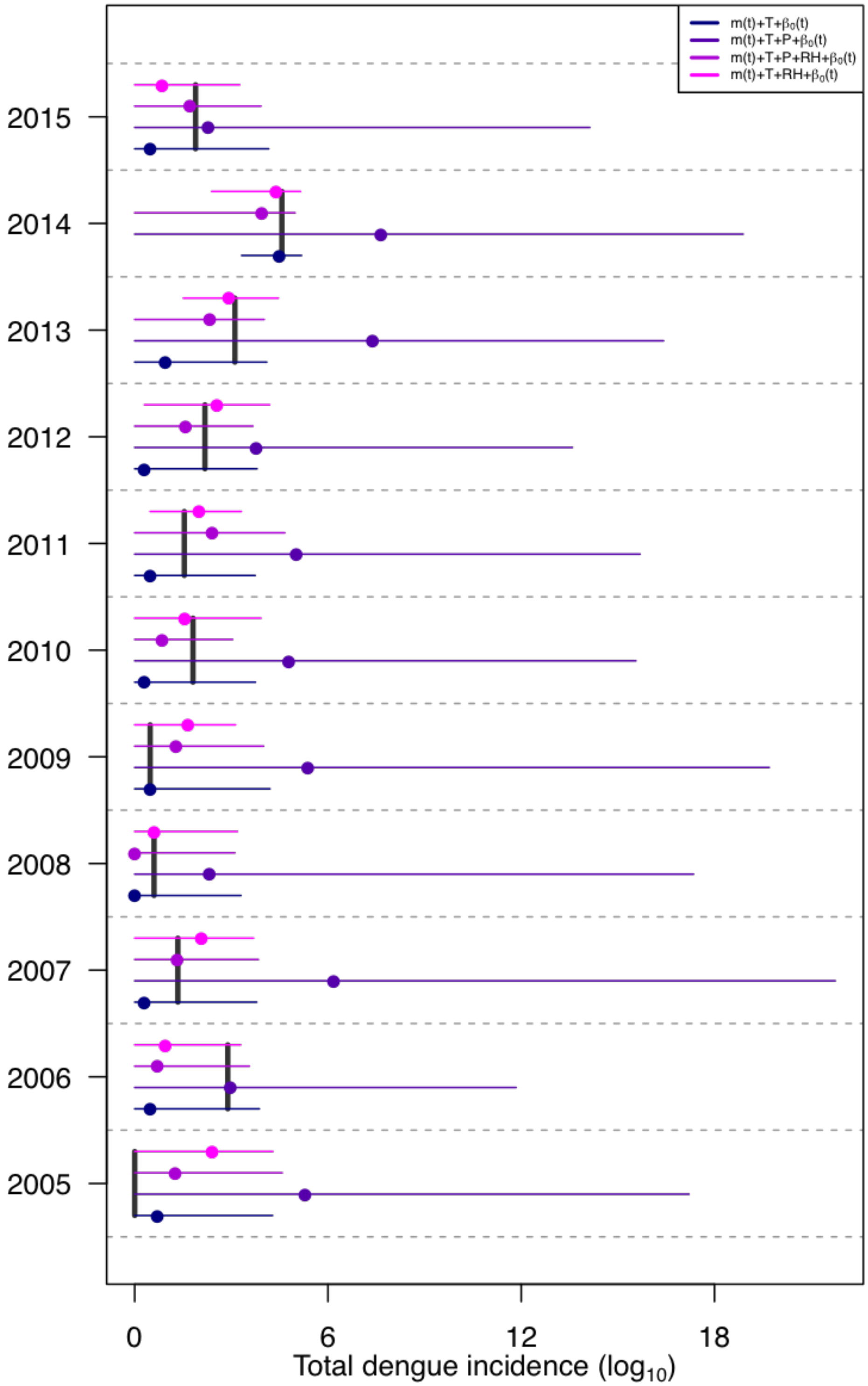

Supplementary Methods 2 Fig. 15. Median and 95% quantile for model simulations and empirical value for simulated total dengue incidence for the primary model and Alternative Models 2-4. Points are median value, horizontal lines are 95% posterior quantile, and vertical line is empirical value.

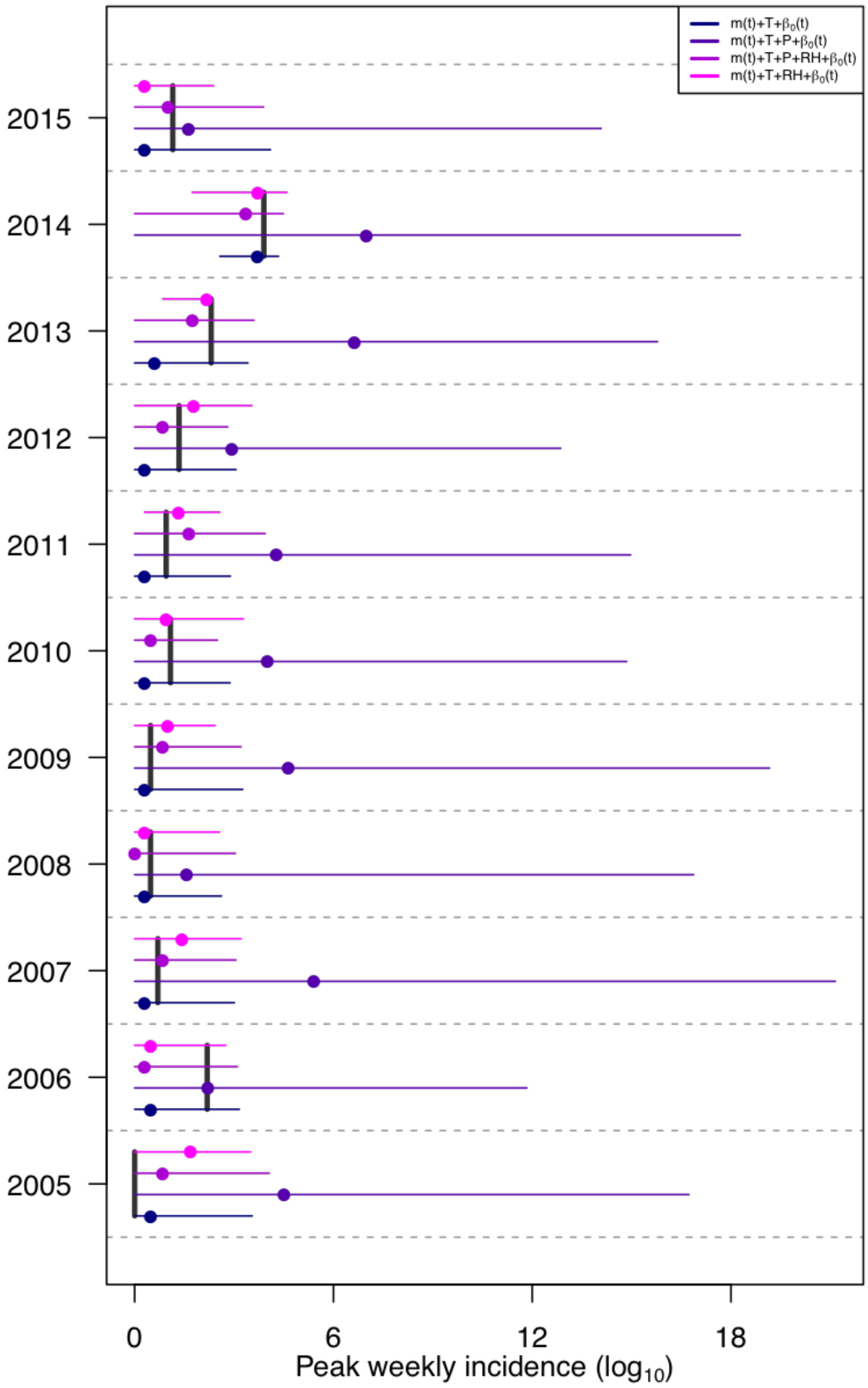

Supplementary Methods 2 Fig. 16. Median and range for model simulations and empirical value for simulated peak weekly incidence for the primary model and Alternative Models 2-4. Points are median value, horizontal lines are 95% posterior quantile, and vertical line is empirical value.

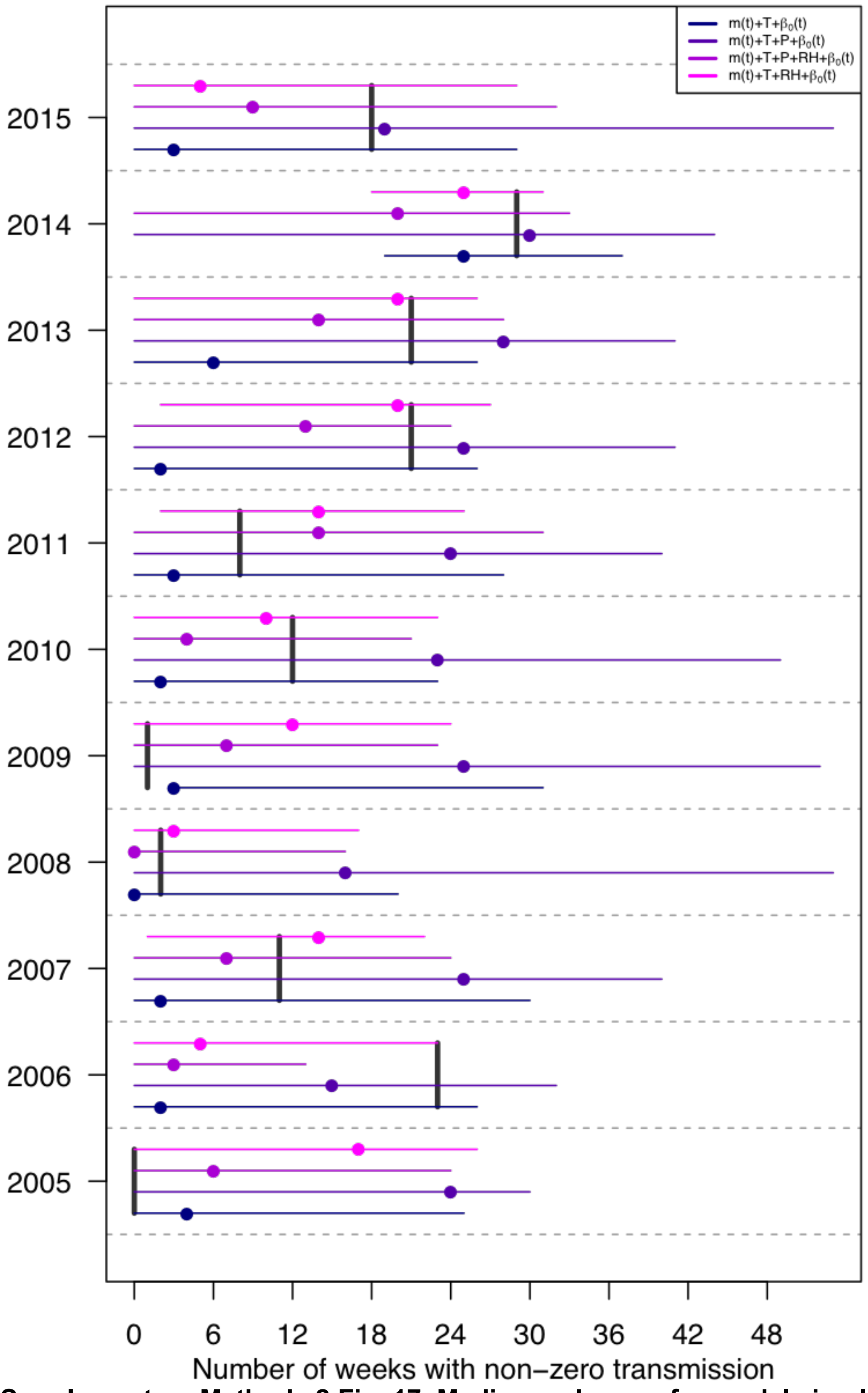

Supplementary Methods 2 Fig. 17. Median and range for model simulations and empirical value for number of weeks with non-zero dengue incidence for the primary model and Alternative Models 2-4. Points are median value, horizontal lines are 95% posterior quantile, and vertical line is empirical value.

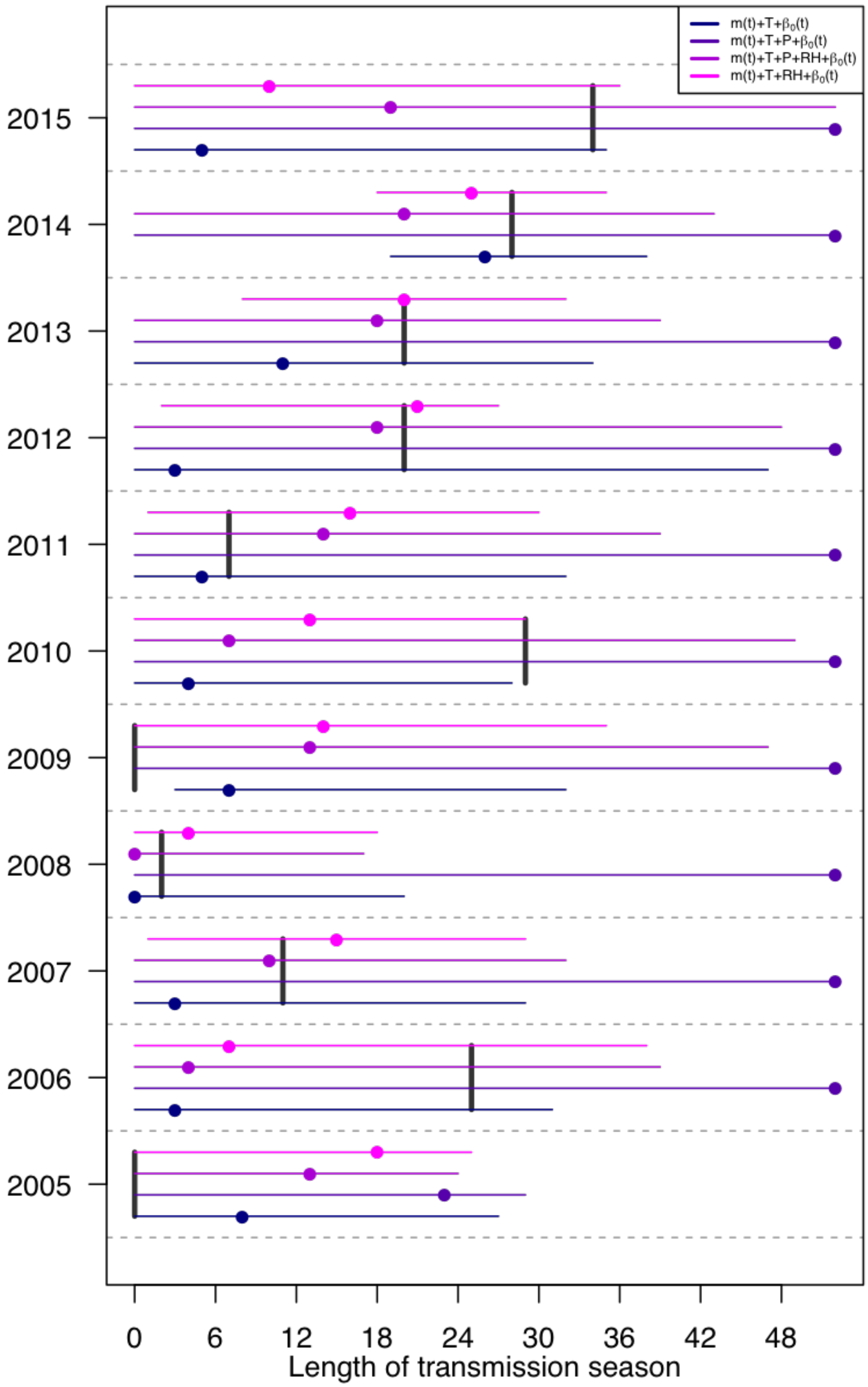

Supplementary Methods 2 Fig. 18. Median and range for model simulations and empirical value for length of transmission season for the primary model and Alternative Models 2-4. Points are median value, horizontal lines are 95% posterior quantile, and vertical line is empirical value. The length of the transmission season was calculated by taking the difference between the first week in which cases presented and the last week in which cases presented.

Supplementary References

1. Xu, L. *et al.* Climate variation drives dengue dynamics. *Proc. Natl. Acad. Sci. U. S. A.* **114,** 113–118 (2016).
2. Sang, S. *et al.* Predicting Unprecedented Dengue Outbreak Using Imported Cases and Climatic Factors in Guangzhou, 2014. *PLoS Negl. Trop. Dis.* **9,** 1–12 (2015).
3. Li, M.-T. *et al.* The Driving Force for 2014 Dengue Outbreak in Guangdong, China. *PLoS One* **11,** e0166211 (2016).
4. Cheng, Q. *et al.* Climate and the Timing of Imported Cases as Determinants of the Dengue Outbreak in Guangzhou, 2014: Evidence from a Mathematical Model. *PLoS Negl. Trop. Dis.* **10,** 1–22 (2016).
5. Cao, Z. *et al.* Individual and interactive effects of socio-ecological factors on dengue fever at fine spatial scale: A geographical detector-based analysis. *Int. J. Environ. Res. Public Health* **14,** (2017).
6. Zhu, G., Liu, J., Tan, Q. & Shi, B. Inferring the Spatio-temporal Patterns of Dengue Transmission from Surveillance Data in Guangzhou, China. *PLoS Negl. Trop. Dis.* **10,** 1–20 (2016).
7. Cheng, Q. *et al.* The interplay of climate, intervention and imported cases as determinants of the 2014 dengue outbreak in Guangzhou. *PLoS Negl. Trop. Dis.* **11,** 1–24 (2017).
8. WorldPop. Population - individual countries. (2016). Available at: http://www.worldpop.org.uk/data/data_sources/.
9. FAO. Global Administrative Unit Layers. Available at: http://www.fao.org/geonetwork/srv/en/metadata.show%3Fid=12691.
10. Kraemer, M. U. G. *et al.* Inferences about spatiotemporal variation in dengue virus transmission are sensitive to assumptions about intra-urban human mobility. *Rev. EPJ Data Sci.*
11. Pya, N. scam: Shape Constrained Additive Models. R package version 1.2-2. (2017).
12. Hawley, W. The biology of Aedes albopictus. *J. Am. Mosq. Control Assoc.* **1,** 1–40 (1988).
13. Higa, Y., Toma, T., Araki, Y., Onondera, I. & Miyagi, I. Seasonal changes in oviposition activity, hatching and embryonation rates of eggs of Aedes albopictus (Diptera: Culicidae) on three islands of the Ryukyu Archipelago, Japan. *Med. Entomol. Zool.* **58,** 1–10 (2007).
14. Ramsay, J. O., Wickham, H., Graves, S. & Hooker, G. fda: Functional Data Analysis. *R Packag. version 2.4.4.* (2014).
15. Mitchell, C. Geographic spread of Aedes albopictus and potential for involvement in arbovirus cycle in the Mediterranean Basin. *J. Vector Ecol.* **20,** 44–58 (1995).
16. Hartig, F., Minunno, F. & Paul, S. BayesianTools: General-Purpose MCMC and SMC Samplers and Tools for Bayesian Statistics. (2017).
