# Supplementary material for: Inter-annual variation in seasonal dengue epidemics driven by multiple interacting factors in Guangzhou, China
Source: Nat Commun. 2019 Mar 8;10:1148. doi: 10.1038/s41467-019-09035-x (PMC6408462; doi:10.1038/s41467-019-09035-x)
Supplement: Supplementary file 3 — Reporting Summary [file 41467_2019_9035_MOESM3_ESM.pdf]

nature research

Corresponding author(s): *Double-blind peer review submissions: write DBPR and your manuscript number here instead of author names.*

Last updated by author(s): *YYYY-MM-DD*

Reporting Summary

Nature Research wishes to improve the reproducibility of the work that we publish. This form provides structure for consistency and transparency in reporting. For further information on Nature Research policies, see Authors & Referees and the Editorial Policy Checklist.

Statistics

For all statistical analyses, confirm that the following items are present in the figure legend, table legend, main text, or Methods section.

n/a	Confirmed	
☒	☐	The exact sample size (*n*) for each experimental group/condition, given as a discrete number and unit of measurement
☒	☐	A statement on whether measurements were taken from distinct samples or whether the same sample was measured repeatedly
☒	☐	The statistical test(s) used AND whether they are one- or two-sided<br>*Only common tests should be described solely by name; describe more complex techniques in the Methods section.*
☐	☒	A description of all covariates tested
☒	☐	A description of any assumptions or corrections, such as tests of normality and adjustment for multiple comparisons
☒	☐	A full description of the statistical parameters including central tendency (e.g. means) or other basic estimates (e.g. regression coefficient) AND variation (e.g. standard deviation) or associated estimates of uncertainty (e.g. confidence intervals)
☒	☐	For null hypothesis testing, the test statistic (e.g. *F*, *t*, *r*) with confidence intervals, effect sizes, degrees of freedom and *P* value noted<br>*Give P values as exact values whenever suitable.*
☐	☒	For Bayesian analysis, information on the choice of priors and Markov chain Monte Carlo settings
☒	☐	For hierarchical and complex designs, identification of the appropriate level for tests and full reporting of outcomes
☒	☐	Estimates of effect sizes (e.g. Cohen's *d*, Pearson's *r*), indicating how they were calculated

Our web collection on statistics for biologists contains articles on many of the points above.

Software and code

Policy information about availability of computer code

Data collection	No software was used for data collection.
Data analysis	We used R Version 3.4.0 to perform analyses.

For manuscripts utilizing custom algorithms or software that are central to the research but not yet described in published literature, software must be made available to editors/reviewers. We strongly encourage code deposition in a community repository (e.g. GitHub). See the Nature Research guidelines for submitting code & software for further information.

Data

Policy information about availability of data

All manuscripts must include a data availability statement. This statement should provide the following information, where applicable:

- Accession codes, unique identifiers, or web links for publicly available datasets
- A list of figures that have associated raw data
- A description of any restrictions on data availability

Data, code, and description of how to replicate the analyses are available on GitHub at https://github.com/roidtman/NatComm_dengue_China.

Field-specific reporting

Please select the one below that is the best fit for your research. If you are not sure, read the appropriate sections before making your selection.

☐ Life sciences ☐ Behavioural & social sciences ☒ Ecological, evolutionary & environmental sciences

For a reference copy of the document with all sections, see nature.com/documents/nr-reporting-summary-flat.pdf

Ecological, evolutionary & environmental sciences study design

All studies must disclose on these points even when the disclosure is negative.

Study description	We fitted a semi-mechanistic model to already-collected data. We performed simulation experiments with this fitted model. We determined these simulation experiments a priori to quantitatively determine the contributions from one factor compared to another factor.
Research sample	Data on locally acquired and imported dengue cases were obtained from the Health Department of Guangdong Provice (http://www.gdwst.gov.cn). We used adult mosquito density and larval density data available from the Guangzhou Center for Disease Control and Prevention (http://www.gzcdc.org.cn). Data on daily temperature were obtained from the China Meteorological Data Sharing Service System (http://data.cma.cn).
Sampling strategy	There was no sampling strategy. All of the data used in this study were passively collected for other reasons and not for our study specifically.
Data collection	Dengue is a statutorily notifiable infectious disease in China, meaning that probable and confirmed dengue cases were diagnosed and reported by local physicians.
Timing and spatial scale	The data used in this study was collected from 2005-2015 on a daily basis in Guangzhou, China.
Data exclusions	We did not exclude any available data.
Reproducibility	Our GitHub site is available in the Data Availability section. Code and data is available here for others to reproduce the study. There were no failed attempts at reproducing the study.
Randomization	Randomization was not relevant to this study as we were not attempting to determine causation from any experiment.
Blinding	Blinding was not relevant to this study.

Did the study involve field work? ☐ Yes ☒ No

Reporting for specific materials, systems and methods

We require information from authors about some types of materials, experimental systems and methods used in many studies. Here, indicate whether each material, system or method listed is relevant to your study. If you are not sure if a list item applies to your research, read the appropriate section before selecting a response.

Materials & experimental systems

n/a	Involved in the study
☒	☐ Antibodies
☒	☐ Eukaryotic cell lines
☒	☐ Palaeontology
☒	☐ Animals and other organisms
☒	☐ Human research participants
☒	☐ Clinical data

Methods

n/a	Involved in the study
☒	☐ ChIP-seq
☒	☐ Flow cytometry
☒	☐ MRI-based neuroimaging
